# Transposon Variants and Their Effects on Gene Expression in *Arabidopsis*

**DOI:** 10.1371/journal.pgen.1003255

**Published:** 2013-02-07

**Authors:** Xi Wang, Detlef Weigel, Lisa M. Smith

**Affiliations:** Department of Molecular Biology, Max Planck Institute for Developmental Biology, Tübingen, Germany; The University of North Carolina at Chapel Hill, United States of America

## Abstract

Transposable elements (TEs) make up the majority of many plant genomes. Their transcription and transposition is controlled through siRNAs and epigenetic marks including DNA methylation. To dissect the interplay of siRNA–mediated regulation and TE evolution, and to examine how TE differences affect nearby gene expression, we investigated genome-wide differences in TEs, siRNAs, and gene expression among three *Arabidopsis thaliana* accessions. Both TE sequence polymorphisms and presence of linked TEs are positively correlated with intraspecific variation in gene expression. The expression of genes within 2 kb of conserved TEs is more stable than that of genes next to variant TEs harboring sequence polymorphisms. Polymorphism levels of TEs and closely linked adjacent genes are positively correlated as well. We also investigated the distribution of 24-nt-long siRNAs, which mediate TE repression. TEs targeted by uniquely mapping siRNAs are on average farther from coding genes, apparently because they more strongly suppress expression of adjacent genes. Furthermore, siRNAs, and especially uniquely mapping siRNAs, are enriched in TE regions missing in other accessions. Thus, targeting by uniquely mapping siRNAs appears to promote sequence deletions in TEs. Overall, our work indicates that siRNA–targeting of TEs may influence removal of sequences from the genome and hence evolution of gene expression in plants.

## Author Summary

Transposable elements (TEs) are selfish DNA sequences. Together with their immobilized derivatives, they account for a large fraction of eukaryotic genomes. TEs can affect nearby gene activity, either directly by disrupting regulatory sequences or indirectly through the host mechanisms used to prevent TE proliferation. A comparison of *Arabidopsis thaliana* genomes reveals rapid TE degeneration. We asked what drives TE degeneration and how often TE variation affects nearby gene expression. To answer these questions, we studied the interplay between TEs, DNA sequence variation, and short interfering RNAs (siRNAs) in three *A. thaliana* strains. We find sequence variation in genes and adjacent TEs to be correlated, from which we conclude either that TEs insert more often near polymorphic genes or that TEs next to polymorphic genes are less efficiently purged from the genome. We also noticed that processes that cause deletions within TEs and ones that silence TEs appear to be linked, because siRNA targeting is a predictor of sequence loss in accessions. Our work provides insight into the contribution of TEs to gene expression plasticity, and it links TE silencing mechanisms to the evolution of TE variation between genomes, thereby linking TE silencing mechanisms to expression plasticity.

## Introduction

While transposable elements (TEs) constitute a large fraction of plant, animal and human genomes [1]–[3], their contribution to genome size can change rapidly during evolutionary time. In some taxa, TEs have been responsible for two-fold differences in genome size that arose over a few million years or less. These rapid fluctuations, which may be due to TEs being either more active or more efficiently deleted in certain species, indicate that control of TEs can differ greatly between closely related plant species [4]–[7]. The balance between TE transpositions and selection against TEs is influenced by factors ranging from mating system to silencing by short interfering RNAs (siRNAs) and chromatin modification. Therefore the control of TE activity and the removal of transposed copies can be considered key factors in the evolution of genomes.

TEs are often regarded as genomic parasites due to the potentially detrimental effects of insertional inactivation of genes and ectopic recombination of DNA [8]. Twenty-four nt long siRNAs are associated with most TEs as part of a ‘double-lock’ mechanism of siRNA-mediated DNA methylation that controls transposition via transcriptional repression, with a reinforcement loop between DNA methylation, histone methylation and siRNAs [reviewed in 9]. siRNAs are a robust proxy for DNA methylation at TEs, with unmethylated TEs generally lacking matching 24 nt siRNAs [10]–[13]. Most plant TEs have cytosine methylation at CG, CHG and CHH sites, but a quarter is unmethylated and a further 15% have atypical methylation patterns. In the TE-dense heterochromatin, DNA methylation can spread about 500 bp into neighboring unmethylated TEs [13]. In the euchromatin, methylation spreads from TEs to approximately 200 bp beyond the siRNA target sites [13], consistent with the effect of siRNAs on expression of proximal genes dissipating by 400 bp [14]. siRNA-targeted, methylated TEs are, on average, located farther away from expressed genes than TEs that are not strongly methylated or associated with siRNAs [13], [15]. As expected from this correlation, siRNA-targeted TEs have more effects on nearby gene expression than those without [14], [15].

Most poorly methylated TEs are short and have few CG dinucleotides [13]. This indicates a progression over evolutionary time from TEs that are active and targeted by siRNA-mediated DNA methylation, to inactive, degenerate relics that have changed through deletions and nucleotide substitutions initiated by deamination of methylated cytosines. These inactive TEs are then no longer targeted by siRNA-mediated DNA methylation.

Presumably because of interference with cis-regulatory elements, *Arabidopsis* TEs reduce the average expression levels of adjacent genes, although the distance over which these effects are noticeable varies between *A. thaliana* and *A. lyrata* [14]. Differences in TEs next to genes contribute to the divergence of gene expression levels between orthologs in these closely related species [14], and gene expression is negatively correlated with the number of nearby siRNA-targeted, methylated TEs [15].

In the selfing species *A. thaliana*, TEs account for only a fifth of the genome [7], [13], [16], making it relatively depauperate of TEs. Given that the *A. thaliana* genome is small relative to other members of the family and that its close relative *A. lyrata*, an outcrosser, contains approximately three times as many TEs [14], deletion of TEs in *A. thaliana* is likely an ongoing, active process. In accordance with this hypothesis, intraspecific polymorphisms and deletions in *A. thaliana* are disproportionately located within TEs and, to a lesser extent, intergenic regions [17]–[19].

A reference-guided assembly approach has been applied to accurately characterize complex sequence variation in several *A. thaliana* accessions [19]. Here, we exploit this information to examine TE variants and their effect on the expression of nearby genes in three divergent accessions. We report that TEs are more likely to be located in polymorphic regions of the genome. Where TEs are present in less polymorphic regions, they also tend to be less polymorphic themselves. Although polymorphic TE variants are less abundantly targeted by siRNAs, uniquely mapping siRNAs targeting polymorphic TE variants are strongly correlated with the TE regions that vary between accessions. These findings suggest a link between the ability to tolerate TE insertions, siRNA-mediated silencing and purging of TEs by deletion.

## Results

### TE variation across the genome

We annotated the sets of genes and TEs in three *A. thaliana* accessions: Col-0, Bur-0 and C24 [19], [20]. For reference accession Col-0, we used the TAIR9 annotation of TEs and protein-coding genes. Excluding centromeric sequences, 21,913 full-length and degenerate TEs and 26,541 genes were considered further. We built genome templates of Bur-0 and C24 from re-sequencing data using the SHORE pipeline [21]. The reference coordinates of TEs and genes were projected onto these genome templates, and variation in TEs and genes was determined based on single nucleotide polymorphisms (SNPs), 1 to 3 bp insertions/deletions (indels) and larger deletions of 4 to 11,464 bp (median 30 bp, mean 113 bp). Larger insertions were not included because of the high false-negative rate [17].

**Figure 1:**
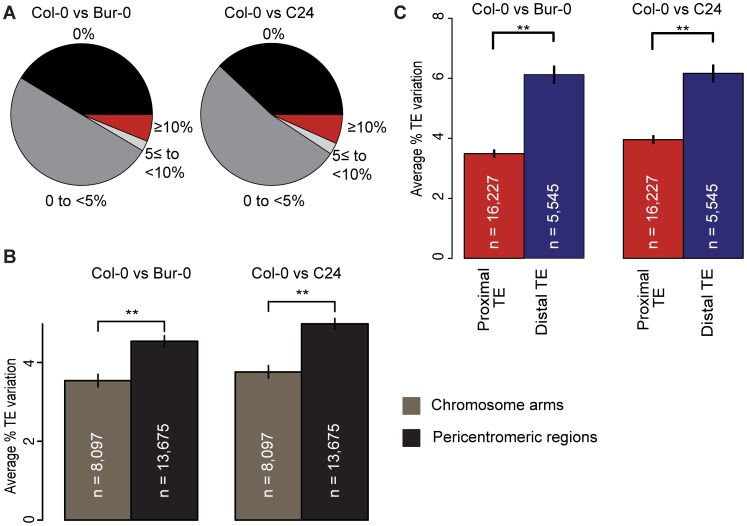
TE variation and its relation to coding gene proximity and genomic region. (a) TE variation between Col-0 and Bur-0 or C24, calculated as the percentage of total TE length that differs between two accessions. (b) for TEs on the chromosome arms vs the pericentromeric regions between Col-0 and Bur-0 or C24. Pericentromeric regions are defined as 8 MB regions flanking the centromeric regions (20). Mann-Whitney U [MWU] test p[Col-0/Bur-0] = 0.001, p[Col-0/C24]<6×10^−5^. ** = p<0.01. Standard errors are shown. (c) Average variation of proximal TEs and distal TEs between Col-0 and Bur-0 or C24 (MWU, p<2×10^−16^ for Col-0 versus Bur-0/C24).

Comparison of polymorphism densities confirmed that coding regions were relatively depauperate of SNPs, indels and large deletions compared to intergenic regions and TEs (binomial test, p[Coding Regions/Intergenic Region] = 0 and p[Coding Regions/TE] = 0 for SNPs, indels or large deletions). Large deletions were significantly over-represented in TEs compared to intergenic regions, while SNPs and indels were not (Figure S1a; binomial test, p[TE/Intergenic Region] = 0 for large deletions). Over 6% of reference TEs differed by at least 10% of total length in each of the two accessions, Bur-0 and C24, compared to Col-0 (Figure 1a and Figure S2). Almost all of this variation, 93%, was due to large deletions (Figure S1b; for distribution of large deletion sizes see Figure S1c). We defined TEs with at least 10% variation by length (SNPs, indels and larger deletions combined), but not completely missing in Bur-0 or C24, as TE variants or VarTEs (please also see Figure S3 for abbreviation definitions). Close to 40% of VarTEs were shared between Bur-0 and C24 (Figure S4a).

TE density is highest in and next to the centromeres, where there are few genes. The fraction of VarTEs and the average level of TE variation were higher in the pericentromeric regions than on the gene-dense chromosome arms (Figure 1b; Mann-Whitney U [MWU] test, p<2×10^−16^ for Col-0 versus Bur-0/C24, Table S1 and Figures S5 and S6). To examine whether gene proximity biases TE variation across the chromosomes, we calculated the distance between TEs and protein-coding genes for Col-0. TEs were separated into two subsets: TEs within 2 kb of any gene, subsequently called proximal TEs, and TEs at least 2 kb away from the closest gene, called distal TEs. Distal TEs were on average more variable than proximal TEs (Figure 1c; Figures S7 and S8; MWU p[Col-0/Bur-0] = 0.001, p[Col-0/C24]<6×10^−5^). Proximity to protein-coding genes may therefore influence TE variation, consistent with TEs closer to genes likely being under stronger selective constraint [15], [22].

The correlation between TE variation and proximity to genes was compared among TE superfamilies [23], [24]. For non-centromeric TEs, LTR retrotransposons were more distal from genes, while no significant difference in distance to genes was observed for other TE superfamilies (Table S2). However, for proximal TEs there were differences among TE superfamilies in distance to genes and, as expected, TE superfamilies that are closer to genes (e.g. CACTA, MITE) were less variable than superfamilies located farther away from genes, e.g. non-LTR retrotransposons (Table S2).

**Figure 2:**
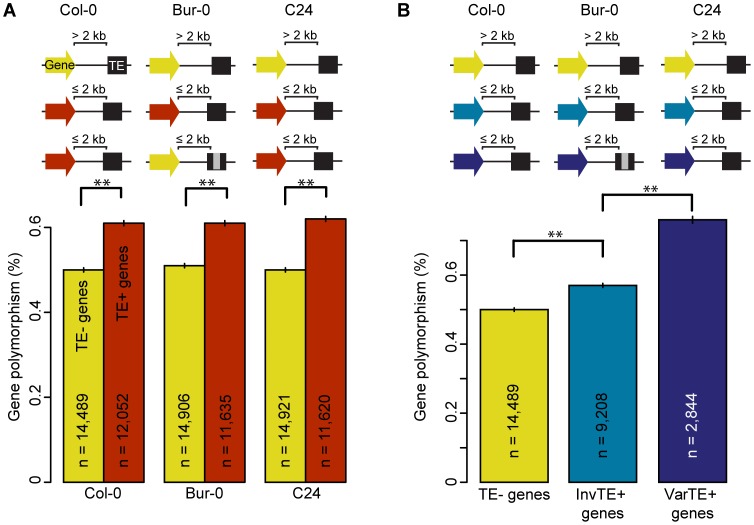
TE presence, variation, and the polymorphism level of proximal genes. (a) Gene polymorphism levels in Bur-0 and C24 for TE− genes (yellow) vs TE+ genes (red). MWU p<2×10^−16^ for Col-0, Bur-0 and C24. Grey regions in the schema represent variations such as deletions. (b) Gene polymorphism levels for TE− genes (yellow), InvTE+ genes (cyan) or VarTE+ genes (navy). MWU p(InvTE+/VarTE+) = 0.005. ** = p<0.01. Standard errors are shown.

To investigate the link between TE and proximal gene variation, we examined whether TE variation and location correlated with the polymorphism level of neighboring genes. We used the small-scale mutations to calculate the polymorphism level of non-centromeric genes. For each accession, genes were separated into two subsets; TE+ genes included genes within 2 kb of a TE and genes with TEs anywhere within the transcribed region, while TE- genes were at least 2 kb from the closest TE (Table S3). To be conservative, any TEs in Bur-0 or C24 with predicted deletions of at least 10% of the reference length were annotated as deleted. TE+ genes were on average more polymorphic than TE− genes in each accession (Figure 2a; MWU p<2×10^−16^ for Col-0, Bur-0 and C24). The same analysis was repeated for 80 resequenced *A. thaliana* accessions [17]; we could confirm the correlations observed with Bur-0 and C24 in these accessions.

Since polymorphism levels vary enormously among gene families, we further investigated whether there is a correlation of TE proximity with gene family using small-scale mutations from the 80 A. *thaliana* accessions (20, 61), and Col-0, C24 and Bur-0. Genes from highly polymorphic families such as those encoding NBS-LRR, F-box and Cytochrome P450s proteins were, on average, closer to TEs in all accessions (Figure S9; distance is negatively correlated with gene polymorphism, Spearman's ρ(Col-0) = −0.11, ρ(Bur-0) = −0.11, ρ(C24) = −0.10; p<2×10^−16^), including a higher proportion of genes having proximal TEs (Figure S10). TEs are therefore either more likely to insert into or near polymorphic genes, or are less efficiently purged from such regions.

To further examine the effects of TE variants on proximal genes, we divided TE+ genes into two subsets: genes where flanking TEs were <10% variant (Invariant TEs: InvTE) among the three accessions (InvTE+ genes), and genes where at least one flanking TE showed ≥10% sequence (VarTE) variation between accessions (VarTE+ genes; Table S3). Three quarters of VarTE+ genes were shared in comparisons between Col-0 and Bur-0 or Col-0 and C24 (Figure S4b). The VarTE+ genes were on average more polymorphic than InvTE+ genes (Figure 2b; MWU p = 0.005), also in the 80 accessions dataset [17]. We conclude that TEs close to genes are less polymorphic, while genes close to polymorphic TEs are themselves more polymorphic.

A correlation between polymorphism levels of TEs and nearby genes is insufficient to address whether this is a direct link as opposed to high directional selection pressure on the genomic region in general. To address this question, we therefore compared the polymorphism level of TEs, the flanking regions and nearby genes. TEs in highly polymorphic regions are themselves more polymorphic than TEs in regions of low divergence (Figure S11a; binomial test, p = 0), with the exception that TEs in highly polymorphic regions with nearby lowly polymorphic genes show a similar level of divergence as TEs in regions of low polymorphism with no coding genes. Moreover, TEs in gene-free regions show significantly higher divergence than TEs within 4 kb of a gene, especially if those genes are less polymorphic. TEs are generally more polymorphic than their flanking sequences (binomial test, p = 0), with the exception of TEs in highly polymorphic regions with lowly polymorphic gene. The results for large deletions (Figure S11b) are consistent with our observation from Figure S1 that large deletions are over-represented in TEs compared to intergenic regions. Notably, there is no significant difference in the level of small-scale mutations between TEs and flanking regions (Figure S11c). Taken together, TE variation through large deletions shows a positive correlation with flanking region polymorphism level, but is also strongly influenced by the conservation and presence/absence of nearby genes. The frequency of large deletions is however generally higher in TEs than in the flanking regions, indicating positive selection for large deletions within TEs.

### TEs, siRNAs, and their effects on expression of adjacent genes

**Figure 3:**
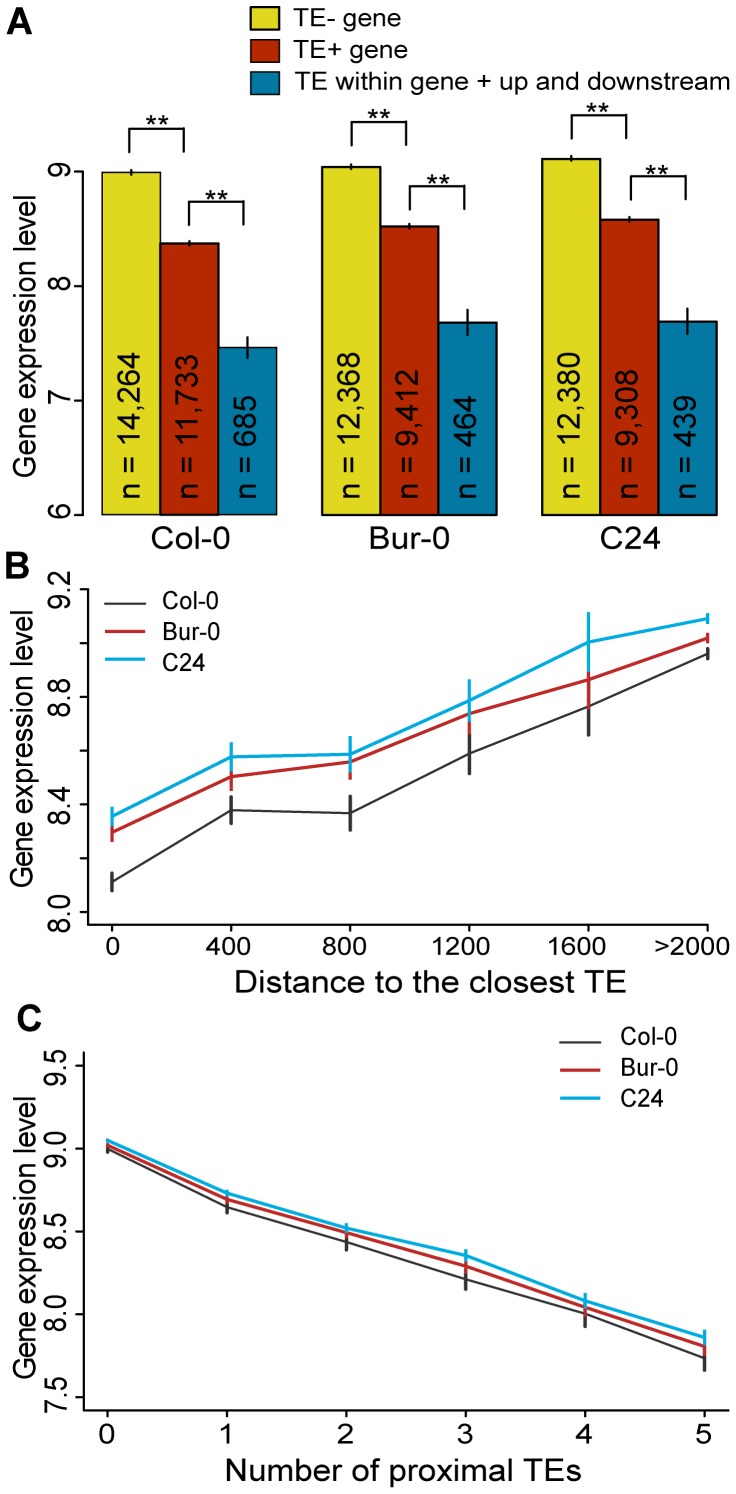
TEs and neighboring gene expression. (a) Average gene expression levels for TE− genes (yellow), TE+ genes (red) and genes where TEs are located simultaneously within, upstream and downstream of the genes (cyan). MWU [TE+/TE−] p<2×10^−16^ for Col-0, Bur-0 and C24, MWU [TE+/TE within gene + up and downstream] p≤2×10^−14^ for Col-0, Bur-0 and C24. (b) Average gene expression as a function of the distance to the nearest TE. Distance was binned into 400 bp windows. A distance of 0 indicates genes that contain a TE. Spearman's ρ(Col-0) = 0.15, ρ(Bur-0) = 0.13, ρ(C24) = 0.13; p<2×10^−16^). (c) Average gene expression as a function of the number of proximal TEs. df = 55, chi-square sums 915, 588 and 553 for Col-0, Bur-0 and C24, respectively, p<2×10^−16^. ** = p<0.01. Standard errors are shown.

Genes that are close to TEs (TE+ genes) tend to have a lower expression average than TE− genes in the Col-0 reference accession [15]. We set out to determine whether this was true for the accessions studied here as well. Gene expression was measured using Affymetrix tiling arrays and RNA extracted from floral tissue of each accession. We considered presence/absence of TEs in the flanking regions of genes, taking into account the number of linked TE insertions and the distance from each gene to the closest TE. We confirmed the reported pattern for Col-0 [15], and found that it applies to Bur-0 and C24 as well. In all three accessions, genes with proximal TEs (TE+ genes) were on average expressed at lower levels than those without proximal TEs (TE− genes; Figure 3a; MWU p<2×10^−16^ for Col-0, Bur-0 and C24). This effect was even stronger if TEs were located simultaneously within, upstream and downstream of the gene (Figure 3a; MWU p≤2×10^−14^ for Col-0, Bur-0 and C24). Moreover, the average expression level of neighboring genes was positively correlated with the distance to the nearest TE (Figure 3b; Spearman's ρ(Col-0) = 0.15, ρ(Bur-0) = 0.13, ρ(C24) = 0.13; p<2×10^−16^), and negatively correlated with the number of proximal TEs (Figure 3c; df = 55, chi-square sums 915, 588 and 553 for Col-0, Bur-0 and C24, respectively, p<2×10^−16^). Thus, gene expression is suppressed by proximal TEs, especially if they are close to the gene and numerous.

Since TE superfamilies may have different effects on proximal genes, we examined gene expression according to the TE superfamily of the closest proximal TE. TE+ genes are expressed differentially depending on the TE superfamily of the proximal TE. TE+ genes with DNA transposons are on average expressed at a higher level compared to TE+ genes surrounded by retrotransposons (Figure S12; MWU, p = 0.02 for Col-0, Bur-0 and C24). However, this is solely due to the higher expression level of genes proximal to CACTA elements. Indeed, we did not find evidence for CACTA TEs having any effect on gene expression (Figure S12, MWU, p(CACTA TE+ genes/TE− genes) = 0.7, 0.6 and 0.8 for Col-0, Bur-0 and C24, respectively), which may explain why they are on average closer to genes than TEs from other families. Within the retrotransposons, LTR retrotransposons are younger on average than non-LTR retrotransposons and have a greater suppressive effect on proximal genes (Table S2; [25]). Therefore TE superfamilies can differ considerably in their effects on proximal genes.

TEs suppress the expression of neighboring genes at least partially through DNA methylation, which in turn is linked to 24-nt long siRNAs [12], [15], [22], [26], [27]. To investigate the influence of siRNAs on TE silencing, we sequenced siRNAs from mixed inflorescence tissue (shoot meristem plus flowers, stages 1–14) of each accession and mapped the reads to all possible positions of the respective genomes without any mismatches. As expected from previous work, the density of siRNAs over TEs was about four times higher than the genome average (Table S4; Figure S13).

**Figure 4:**
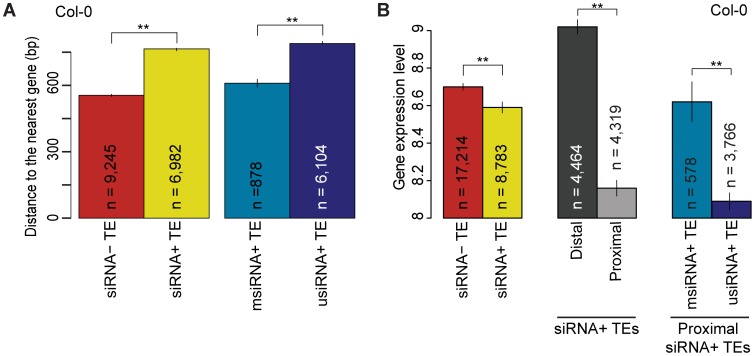
Relationship of TE siRNA–targeting to distance from genes and its effect on gene expression in Col-0. (a) Average distance of siRNA− (red) and siRNA+ (yellow) proximal TEs to the nearest gene. For siRNA+ proximal TEs, distance to the closest gene is compared between msiRNA+ TEs (cyan) and usiRNA+ TEs (navy). MWU [siRNA+/siRNA−] p<2.2×10^−16^ for Col-0, Bur-0 and C24. MWU [msiRNA/usiRNA] p[Col-0]<2×10^−16^, p[Bur-0] = 6×10^−13^ and p[C24] = 2×10^−6^. (b) Average expression level of genes when neighboring TEs are siRNA− (red) or siRNA+ (yellow). For siRNA+ TEs, average gene expression levels are given for when the nearest TE is distal (greater than 2 kb from gene; dark gray) or proximal (within 2 kb; light gray). For genes with proximal siRNA+ TEs, expression levels were further compared between msiRNA+ TEs (cyan) and usiRNA+ TEs (navy). See Figure S12 for Bur-0 and C24. MWU [siRNA+/siRNA−] p[Col-0] = 0.0001, p[Bur-0] = 0.002, p[C24] = 2×10^−6^. MWU [siRNA+ distal/proximal] p<2×10^−16^ for Col-0, Bur-0 and C24. MWU [msiRNA/usiRNA] p[Col-0] = 3×10^−6^, p[Bur-0] = 5×10^−5^, p[C24] = 0.01). ** = p<0.01. Standard errors are shown.

We have reported before that siRNA-targeted TEs are more effective in suppressing expression of neighboring genes than are non-siRNA-targeted TEs, and that they are farther from genes [15]. We determined whether this held true in the current, more comprehensive dataset. If at least one 24-nt siRNA mapped to a TE it was labeled as siRNA+ (Table S5). siRNA+ and siRNA− TEs were overall similar in number, but retrotransposons were targeted by siRNAs more frequently than DNA transposons (Figure S14; binomial test, p = 0 for Col-0, Bur-0 and C24). siRNA+ TEs were farther from genes (Figure 4a; Figure S15a; MWU p<2.2×10^−16^ for Col-0, Bur-0 and C24), and this bias was consistent among TE superfamilies (Figure S16). To examine the effects of siRNA-targeting on the expression of flanking genes, we classified genes by whether the nearest TE was siRNA+ or siRNA− (Table S5). In each accession, genes flanked by siRNA+ TEs had lower average expression levels than genes with adjacent siRNA− TEs (Figure 4b; Figure S15b; MWU p[Col-0] = 0.0001, p[Bur-0] = 0.002, p[C24] = 2×10^−6^). The effect of suppression was stronger if the closest siRNA+ TE was within 2 kb of the gene (Figure 4b; Figure S15b; MWU p<2×10^−16^ for Col-0, Bur-0 and C24). Therefore, as found previously for Col-0, siRNA-targeting of TEs represses nearby genes and TEs that are close to genes are less likely to be targeted by siRNAs, either due to stronger selection for deletion of siRNA-targeted TEs close to genes or selection against siRNA-targeting of these TEs.

Because siRNAs that map to unique positions in the genome (usiRNAs) correlate more closely with DNA methylation than siRNAs that map to multiple positions (msiRNAs; [12]), we investigated whether usiRNAs and msiRNAs target TEs differentially, and how usiRNA− and msiRNA-targeted TEs might affect the expression of nearby genes. All TEs with at least one usiRNA were labeled as usiRNA+ (Table S5). In both Bur-0 and C24, over 83% of siRNA+ TEs were usiRNA+, similar to what has been reported for Col-0 [14]. usiRNA+ TEs were farther away from genes than msiRNA+ TEs (Figure 4a; Figure S15a; MWU p[Col-0]<2×10^−16^, p[Bur-0] = 6×10^−13^ and p[C24] = 2×10^−6^). We also observed that the average expression level of genes within 2 kb of usiRNA+ TEs was lower than the expression of genes within 2 kb of msiRNA+ TEs (Figure 4b; Figure S15b; MWU p[Col-0] = 3×10^−6^, p[Bur-0] = 5×10^−5^, p[C24] = 0.01). Therefore, even though TEs targeted by usiRNAs and msiRNAs are on average farther from genes, they more strongly reduce expression of proximal genes compared to TEs targeted by only msiRNAs. Overall, we confirmed that siRNA+ TEs, especially usiRNA+ TEs, suppress neighboring gene expression, consistent with a trade-off between reduced TE mobility and deleterious effects on neighboring gene expression [14], [15].

### Links between variation in TEs, siRNA–targeting, and gene expression differences

**Figure 5:**
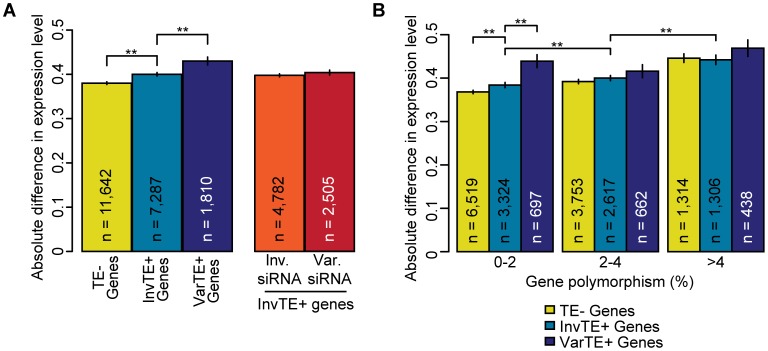
TE variation, siRNA–targeting, and differences in proximal gene expression. (a) Average absolute difference in gene expression for TE− genes (yellow), InvTE+ genes (cyan) and VarTE+ (navy) genes. MWU p[TE−/TE+]<2×10^−16^, p[InvTE+/VarTE+] = 2×10^−5^. Expression divergence is also shown for InvTE+ genes divided by whether the proximal TEs are invariably (orange) or variably (red) targeted by siRNA. (b) TE− genes (yellow), InvTE+ (cyan) genes and VarTE+ (navy) genes were divided into subgroups depending on their polymorphism levels. Genes were binned by polymorphism levels into 0–2%, 2–4% and >4% groups. The average absolute change in expression level for each subgroup of genes is shown. ** = p<0.01. Standard errors are shown.

If TEs suppress the expression of adjacent genes, presence of gene-proximal TEs in the different accessions should be associated with differences in expression levels of proximal genes. We found that expression of TE− genes varied less between accessions than TE+ genes, and further that expression varied less between genes proximal to invariant TEs (InvTE+ genes) than genes proximal to variant TEs (VarTE+ genes; Figure 5a; MWU p[TE−/TE+]<2×10^−16^, p[InvTE+/VarTE+] = 2×10^−5^). However, because TEs, and especially VarTEs, are found more often next to polymorphic genes, these conclusions could be confounded by correlated differences in genic polymorphisms. We therefore classified genes based on the extent of sequence variation (Table S6). Regardless of degree of genic polymorphism, VarTE+ genes were the ones that varied most in expression between accessions (Figure 5b), indicating that TE variation increases variance in gene expression.

We next determined whether differential siRNA-targeting influences gene expression. To remove the potentially confounding effects of variation in TEs themselves, we focused on InvTE+ genes and grouped these based on whether siRNAs for the adjacent TE could be detected in either all or none of the three accessions, or whether accessions differed in siRNA-targeting of the adjacent TE. We found that while variation in siRNA-targeting increased expression differences between accessions, this increase was not statistically significant (Figure 5a). It should be noted that in our analysis we could not distinguish between the effects of differential siRNA-targeting and any perturbations of cis-regulatory sequences.

Since each TE that differs in presence/absence or each siRNA-targeting variant between accessions represents a natural mutagenesis experiment, this offers an opportunity to study the effects on individual genes, to confirm the inferences drawn from averaging over all genes. We selected siRNA+ TE+ genes in Col-0 that are siRNA− TE+ or TE− in Bur-0 or C24 and tested for differential expression between Bur-0 or C24 and Col-0. To remove the potential confounding effect of genic polymorphism, we excluded genes with a polymorphism level greater than 2%. Overall 706 genes were retained for this analysis. The effect of siRNA-targeting on gene expression was further verified by comparing expression profiles among wild-type, *rdr2-1* and a *ddc* (*drm1drm2cmt3*) DNA methyltransferase triple mutant [28]. Fifteen genes out of 706 showed significant up-regulation (top 5% ranking) in Bur-0 or C24 and in at least one of the RNA silencing mutants (Table S7). Although not statistically significant, this observation is consistent with siRNA-targeting and TE presence affecting gene expression. Moreover, it is likely an underestimate of TE effects on gene expression, given our stringent selection criteria.

### siRNA–targeting and TE evolution

**Figure 6:**
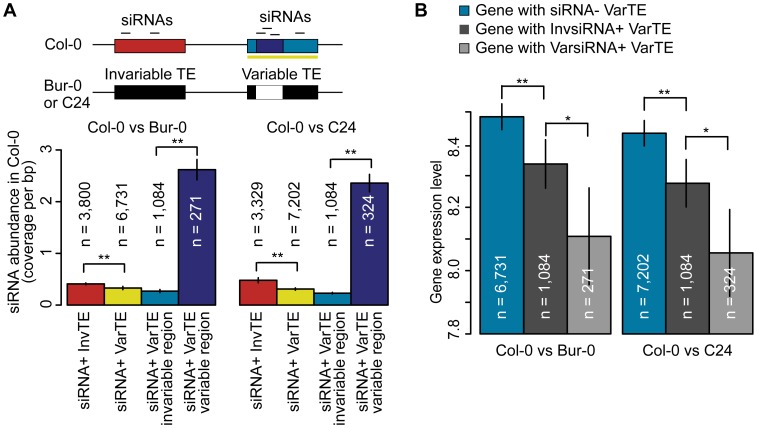
siRNA–targeting of VarTEs and the effect on proximal gene expression. (a) Upper panel depicts siRNA-targeting of variable and invariable regions of VarTEs defined between Col-0 and Bur-0 or C24. Lower panel shows abundance of Col-0 siRNA in siRNA+ InvTEs (red) and siRNA+ VarTEs (yellow) between Col-0 and Bur-0 or C24. Within siRNA+ VarTEs, the abundance of Col-0 siRNA was compared between invariable (cyan) and variable regions (navy). MWU [InvTE/VarTE] p<2×10^−16^ for Col-0 versus Bur-0/C24. MWU [variable/invariable regions of VarTEs] p[Col-0/Bur-0] = 1×10^−5^, p[Col-0/C24]<2×10^−16^ (b) VarTE+ genes were divided into subgroups based on whether the closest proximal TE was siRNA− (cyan), InvsiRNA+ (dark gray) or VarsiRNA+ (light gray). The average expression level of each gene group is shown. MWU [siRNA− VarTE+/InvsiRNA+ VarTE+] p[Col-0/C24] = 0.01, p[Col-0/Bur-0] = 0.01; MWU [siRNA− VarTE+/VarsiRNA+ VarTE+] p[Col-0/C24] = 9×10^−5^, p[Col-0/Bur-0] = 0.003; MWU [VarsiRNA+ VarTE+/InvsiRNA VarTE+] p[Col-0/C24] = 0.01, p[Col-0/Bur-0] = 0.04; * = p<0.05, ** = p<0.01. Standard errors are shown.

Because siRNA+ TEs suppress neighboring gene expression particularly efficiently, we asked whether targeting of different regions of TEs was reflected in the expression of adjacent genes. We first investigated whether invariant and variant TEs (InvTEs and VarTEs) differed in siRNA-targeting, normalized by TE length, and whether there were differences between invariable and variable regions of VarTEs (Figure 6a; Table S8). Fewer siRNAs mapped to siRNA+ VarTEs than to siRNA+ InvTEs (Figure 6a; MWU p<2×10^−16^ for Col-0 versus Bur-0/C24), but there were more siRNAs in variable regions than invariable regions of siRNA+ VarTEs in Col-0 (Figure 6a; MWU p[Col-0/Bur-0] = 1×10^−5^, p[Col-0/C24]<2×10^−16^). Furthermore, usiRNAs were overrepresented in variable regions (binomial test, p[Col-0/Bur-0] = 7×10^−18^, p[Col-0/C24] = 0), while msiRNAs were biased towards invariable regions (p[Col-0/Bur-0] = 1×10^−6^, p[Col-0/C24] = 0). Therefore, usiRNAs strongly correlate with variability of TE sequences and are over-represented in the variable regions of variant TEs.

This finding raised the question whether TE regions that varied between accessions and were targeted by siRNAs had a particularly large effect on expression of adjacent genes. We therefore separated Col-0 genes within 2 kb of variable TEs into three subsets: genes next to siRNA− VarTEs (siRNA− VarTE+ genes); genes next to VarTEs with an siRNA-targeting bias towards invariable TE regions (InvsiRNA+ VarTE+ genes); and genes next to VarTEs with an siRNAs targeting bias towards variable TE regions (VarsiRNA+ VarTE+ genes; Table S8). As expected, siRNA− VarTE+ genes had a higher average expression level compared to InvsiRNA+ VarTE+ genes (Figure 6b; MWU p[Col-0/C24] = 0.01, p[Col-0/Bur-0] = 0.01) or VarsiRNA+ VarTE+ genes (MWU p[Col-0/C24] = 9×10^−5^, p[Col-0/Bur-0] = 0.003). The InvsiRNA+ VarTE+ genes, however, were expressed on average more highly than the VarsiRNA+ VarTE+ set (MWU p[Col-0/C24] = 0.01, p[Col-0/Bur-0] = 0.04). This indicates that gene suppression by neighboring TEs may not only be influenced by siRNA presence or absence at the TEs, but may also depend on which TE regions are targeted by siRNAs. We speculate that siRNA-targeting of particular TE regions suppresses the expression of nearby genes to such an extent that there is significantly higher selection pressure for these regions to be excised or mutated. Alternatively, due to the skew of usiRNA mapping towards variable regions, and the greater correlation between usiRNAs and TE methylation, the lower expression level of VarsiRNA+ VarTE+ genes may reflect a higher degree of epigenetic silencing of these elements compared to InvsiRNA+ VarTE+ genes.

## Discussion

TEs constitute the majority of DNA in many plant genomes [2], [3]. Evolutionary dynamics vary among TE types and they are affected, for example, by species demography and mating system [29]. A number of measures counteract the proliferation of TEs including TE silencing and removal. Because TE deletions via illegitimate recombination and unequal intra-strand homologous recombination are common [30]–[33], it is important to understand how changes in TE composition affect nearby gene expression. We have studied the interactions of TE variants, genic polymorphism, gene expression, and siRNA-targeting in *Arabidopsis thaliana*. We have shown that there is substantial variation in TEs between accessions primarily through large deletions, with invariant TEs on average closer to genes than variant TEs. We have confirmed that gene expression is positively correlated with distance to the nearest TE, and negatively correlated with the number of proximal TEs. While variation within a TE has some effect on the expression of adjacent genes, genes close to TEs are also on average more polymorphic than those that are not. Perhaps our most interesting observation is the increased usiRNA-targeting in TE regions that are variable between accessions compared to TE regions that are invariant.

### TE variation between accessions

TEs may be prevented from reaching fixation within a population through negative selection, especially for gene-proximal, methylated TEs [13], [15], [34]. Therefore, it is perhaps unsurprising that TEs are over-represented in analyses of structural variants among accessions and between species [17], [18], [35], [36], and that a recent comparison of 80 *A. thaliana* genomes reported evidence of structural variation in 80% of TEs [17]. Similarly, Hollister and Gaut [15] found that 44% of over 600 TE insertions were polymorphic among 48 accessions. Since most TEs in *A. thaliana* are relatively old [7], the simplest way to explain these patterns is ongoing deletion of TEs, which is also consistent with TEs in *A. thaliana* being on average farther from genes than in the closely related but outcrossing *A. lyrata* [7]. This may, however, be too simplistic an explanation as non-LTR retrotransposons are skewed towards an older insertion distribution than LTR retrotransposons [25], even though they are not significantly more variable (Table S2). While TE presence/absence polymorphisms in different accessions have been previously characterized [17], we have shown that there is substantial sequence variation in about 6% of TEs when comparing accessions (Figure 1a). These TE variants are equally distributed throughout the genome (Figure 1b).

### TE effects on nearby genes

TEs can affect the expression of proximal genes via mechanisms including disruption of promoter sequences, reduction of transcription through the spread of epigenetic silencing [13], or read-though antisense transcription [37]. Often TEs suppress the expression of proximal coding genes [15], [22], [38] however, TEs can also introduce new promoter sequences, leading to up-regulation of proximal genes [37]. In both plants and animals, TE-derived sequences have been recruited to form regulatory sequences and have contributed to coding regions [8], [39]–[42].

Methylated TEs suppress expression of proximal genes in *A. thaliana*, regardless of insertion upstream or downstream of the coding region. Purifying selection is therefore greatest for methylated TEs proximal to genes [15]. Notably, the effects of siRNAs on expression of proximal genes can only be detected up to 400 bp [14], while measurable TE effects extend to 2 kb [14]. This supports the assertion that TEs either directly affect gene expression by disruption of positive regulatory sequences, or otherwise act through DNA structure and epigenetic marks to affect genes over longer distances.

We found that TEs that with variable siRNA-targeting do not affect proximal genes more strongly than TEs that are targeted in all three accessions (Figure 5). It is possible that siRNA-targeting varies independently of TE sequence variation, as observed recently for DNA methylation [43], and that such TEs mask more subtle differences between the TE classes examined. However, the region of the TE targeted by siRNAs does seem to matter, with siRNA-targeting of TE sequences within an accession that are variant/absent in other accessions showing a greater suppression of proximal genes (Figure 6). This agrees with the observation that genes close to usiRNA-targeted TEs have a lower expression average than those close to msiRNA-targeted TEs, and that usiRNAs are over-represented in the variable regions of transposons. A recent study of hybrids between parents of different ploidy found that a reduction in 24 nt siRNAs is associated with up-regulation of more TE-associated genes than when there is no significant change in siRNA levels [44]. This result supports the hypothesis that siRNAs, or linked epigenetic changes, can affect the expression of nearby genes, with deletion of the siRNA-targeted regions alleviating repression of adjacent genes.

While TEs in the euchromatin are often found close to genes, methylated TEs are underrepresented upstream of genes, likely because changes in the promoter more easily affect gene expression than variation in the 3′ region [13]. In agreement, methylated TEs have a skewed distribution, with older elements farther from genes, but unmethylated TEs do not show such a bias [15]. In a comparison of humans and chimpanzees, TE insertion site preference appears to be the main cause for TEs being found more often in the vicinity of genes with increased interspecific expression variation [45]. This is reminiscent of what we have observed, with additive effects of polymorphism, TE presence and TE variance on the variability of orthologous gene expression (Figure 2 and Figure 4). In a comparison of two rice subspecies, TE presence/absence polymorphisms were also found to be underrepresented in SNP deserts [35]. There are several possible explanations for these observations: some genomic regions may suffer from generally elevated mutation rates TEs near highly conserved genes are more efficiently purged; or TE integration into more mutable genomic regions is favored. In the latter case, new mutations may destabilize DNA packing and facilitate TE insertions, similar to the TE insertion preference for transcribed genomic regions [42].

### TE evolution through silencing and deletions

**Figure 7:**
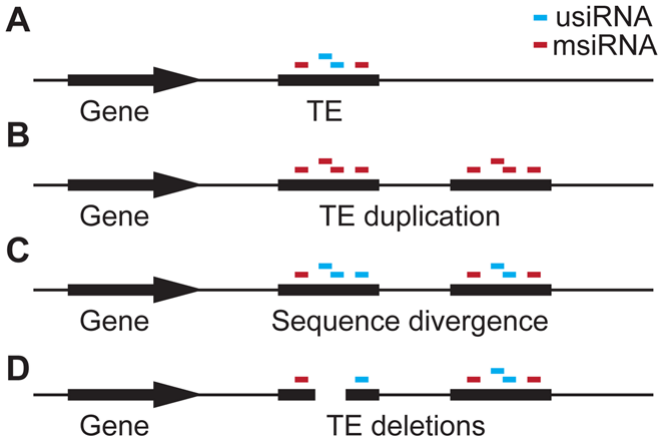
Hypothesis for the role of siRNA–targeting in TE evolution. (a) A gene with an adjacent TE targeted by siRNAs that are either unique to this TE (usiRNAs) or that are shared with multiple locations in the genome (msiRNAs). (b) Duplication of the TE causes all usiRNAs to become msiRNAs. (c) Sequence divergence between the duplicated TEs, e.g. through deamination of methyl-cytosines, which causes C:T transition mutations. As a consequence, msiRNAs are converted to usiRNAs again. (d) TE regions that are enriched for siRNAs, especially usiRNAs, are deleted, reducing the effect of the TE on adjacent genes.

With our observation of TE deletions correlating with siRNA-targeting, we can expand the current model for TE evolution [15]. Our model starts with the duplication of a TE that is already present and targeted by siRNAs within the genome (Figure 7a and 7b), leading to all siRNAs produced by and targeting the original TE now being multiply-mapping siRNAs (msiRNAs). As the two copies of the duplicated TE gain mutations (enhanced by deamination of methylated cytosines), uniquely-mapping siRNAs (usiRNAs) are produced in addition to msiRNAs (Figure 7c). Hollister and colleagues [14] noted that usiRNA-targeting increases with TE age, while msiRNA-targeting decreases, and that TEs are expressed at lower levels when also targeted by usiRNAs. Furthermore, usiRNAs are more closely correlated with DNA methylation than are msiRNAs [12] and they are expressed at higher levels than msiRNAs [14]. With usiRNAs, the duplicated TEs will therefore be more effectively silenced, probably with a concurrent increase in methylation, a further reduction in the expression level of proximal genes, and thus increased selection against the TEs.

usiRNA-targeting may then facilitate TE inactivation through preferential deletion of usiRNA-targeted regions (Figure 6 and Figure 7d). This may be actively promoted by the usiRNAs and attendant epigenetic marks, in a mechanism analogous to the siRNA-guided removal of “internal eliminated sequences” including TEs in *Tetrahymena* [46], [47]. In favor of such a scenario, small deletions within TEs have been shown to occur more frequently than ectopic recombination events at the LTRs [31], [48]. Ectopic recombination appears to be less important for TE elimination in *A. thaliana*, as TE density and recombination rate are not correlated in this species [48], and because ectopic recombination is lower in homozygotes [49]. No matter what the mechanism, deletions within TEs would reduce selection pressure by removing usiRNA target sites, inactivating TEs so they are no longer transposition-competent, and relieving proximal gene repression.

In apparent contrast to the majority of TEs, some are under positive selection [50], [51], and TEs can also contribute to new regulatory networks [52]. Our model is only appropriate for TEs under neutral or negative selection. Modeling of TE dynamics suggests that transposition events occur in a cyclical manner [53], [54], with some activation events creating new favorable genetic variants. One such example is provided by transposition of a TE that is induced upon heat stress in genetic backgrounds impaired in siRNA biogenesis confers heat-responsiveness to proximal genes [55].

### Conclusions

We have exploited high-quality genome information from multiple accessions of a single species to study the effects of TE variation on proximal gene expression. We discovered a link between siRNA-targeting and TE variation that illuminates how epigenetic mechanisms may help to shape genomes, but several questions remain: Do usiRNAs directly facilitate TE deletions or do they act indirectly through differences in selection for deletions? Are TE deletions in other species also associated with regions of increased usiRNA-targeting? And do species differ in the rate of TE deletion via this mechanism? Because of the rarity of TE deletions, this is a challenging process to dissect. Genomes with a large fraction of TEs, such as those of many crop plants, might therefore prove more tractable systems for studying mechanism of TE removal than the TE poor *A. thaliana* genome.

## Methods

### Annotation of genes and TEs in Col-0, Bur-0, and C24

We extracted positions of genes and TEs from the *A. thaliana* Col-0 genome sequence TAIR version 9 from http://www.arabidopsis.org. We excluded genes and TEs within the centromeric regions [56]. To define gene and TE sets in Bur-0 and C24, we built genome templates using published Illumina paired-end reads of Bur-0 and C24 [19]. We used the SHORE pipeline [21] to align the reads to the Col-0 reference genome and extracted the consensus sequences as genome templates by calling bases with quality>24, support>6, concordance>0.7 and average hits = 1. We then applied a naïve projection of the coordinates of genes and TEs from Col-0 onto the genome templates to define the gene and TE sets of Bur-0 and C24. SHORE was also used to detect genomic variations by calling SNPs, small (1–3 bp) insertions/deletions and larger deletions from the genome templates of Bur-0 and C24 compared to the Col-0 genome using the same parameters for quality control. The distance between TEs and genes in Bur-0 and C24 was estimated from Col-0 using the annotated TE and gene coordinates, and adjusted to account for insertions and deletions between TEs and genes.

### Comparison of polymorphism densities

For each polymorphism type (i.e., SNPs, small indels, and large deletions), we compared the densities pairwise across coding regions, intergenic regions and TEs. To test whether a higher density was significant in a particular genomic region (e.g. TE) compared to others (e.g. coding region), a cumulative binomial probability distribution was applied:
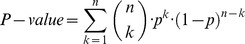

*p* is the polymorphism density in coding regions, and *k* and *n* are the total number of polymorphic sites in TEs and the total length of TEs, respectively.

We calculated gene polymorphism levels as the fraction of genic region containing small-scale variations in at least C24 or Bur-0, or one of the 80 *A. thaliana* accessions [17]. Genes with more than 20% zero sequencing coverage or no base calls among 80 accessions were excluded from the analysis.

4 kb 5′ and 3′ flanking regions for each TE were extracted. For each flanking region (FR), or genic regions (GR) within the FR, small-scale mutations and large deletion polymorphisms between Col-0 and Bur-0/C24 were calculated. Using all mutations, the polymorphism levels of TEs, FRs and GRs were ranked. A threshold of 50% was used to split FRs and GRs into high or low polymorphism datasets and thereby classify the TEs by genomic environment. The polymorphism levels of the FRs were calculated in 200 bp bins for each group of TEs, with binomial tests to compare polymorphism levels between TEs and FRs, and between different TE groups.

### Gene expression

Inflorescences (meristem and flowers up to stage 14) were pooled from five plants of each accession grown at 23°C. Triplicate samples were collected between 7 and 8 hours into a 16 hour light cycle. RNA was extracted using the Qiagen (Hilden, Germany) Plant RNeasy Mini kit. Each biological replicate was analyzed with Affymetrix (Santa Clara, CA, USA) tiling 1.0R arrays and the data were processed according to published methods [57], [58]. Tiling array probes that were polymorphic for C24 or Bur-0 were removed from the dataset for the affected accession(s). For gene expression estimates, ≥70% and at least 3 probes had to be present; all other genes were not considered.

Tilling array data from *Arabidopsis* Col-0 and the RNA silencing mutants *rdr2-1* and *ddc* (*drm1-1;drm2-2;cmt3-11*) mutants were downloaded from GEO (GSE12549; [28]) and processed according to published methods [57], [58]. Expression level changes for each dataset were estimated by fold-change differences between Bur-0/C24 and Col-0, and between the RNA silencing mutants and wild type Col-0. Background distributions of fold-change were calculated and genes, with a fold-change exceeding a one-sided 95% quantile in each dataset were considered as significantly up-regulated in Bur-0/C24 or the mutants.

### siRNA analyses

The siRNA datasets have been published [19] (GEO accession number GSE24569). We mapped the 24-nt siRNA reads onto both strands of the genome templates (see below) and the TEs of Col-0, Bur-0 and C24, respectively, using the *Vmatch* package (http://www.vmatch.de). Only reads with perfect matches were considered.

### Comparison of usiRNA– and msiRNA–targeting

The statistical significance of over-representation of usiRNAs or msiRNAs within the variable regions of siRNA+ VarTEs in comparison to all siRNAs was tested using the cumulative binomial probability distribution given above. *p*, expected frequency, is the ratio between the number of siRNAs mapped to the variable regions the total number of siRNAs mapped to any region of siRNA+ VarTEs, and *n* and *k* are the total number of usiRNAs/msiRNAs mapped to any region and the number of usiRNAs/msiRNAs mapped to the variable regions, respectively.

### Determination of InvsiRNA+ and VarsiRNA+ VarTEs

We defined an siRNA+ VarTE as either InvsiRNA+ or VarsiRNA+ if siRNAs are overrepresented in the invariable regions and variable regions, respectively. For siRNA+ VarTEs that contain siRNAs in both variable and invariable regions, we employed the cumulative binomial probability distribution described above to test whether siRNA-targeting shows statistically significant bias towards variable or invariable regions. For each siRNA+ VarTE, p in the formula above is the abundance of siRNA-targeting at the TE. To test the bias towards variable regions, n and k represent the genomic length of variable regions and the number of siRNAs targeting variable regions, respectively. Similarly, to test the bias towards invariable regions, n and k represent the genomic length of invariable regions and the number of siRNAs targeting invariable regions, respectively. P-values were adjusted for multiple hypothesis testing with the Benjamini-Hochberg method to control for a false discovery rate of 5% [59].

### Data deposition

The siRNA and microarray data reported in this paper have been deposited in the National Center for Biotechnology Information Gene Expression Omnibus (NCBI GEO) (http://www.ncbi.nlm.nih.gov/geo/) under accession numbers GSE24569 and GSE24669. The genome assemblies are available from http://1001genomes.org/projects/MPIWang2012/ while the transposable element annotations for Bur-0 and C24 are available from Dryad under doi 10.5061/dryad.8674d.

## Supporting Information

Figure S1 TE variation in Col-0, Bur-0 and C24. (a) Polymorphism densities for coding regions, intergenic regions and TEs according to polymorphism type. Binomial tests: p[Coding Regions/Intergenic Region] = 0 and p[Coding Regions/TE] = 0 for SNPs, indels or large deletions); p[Intergenic Regions/TE] = 0 for large deletions, (b) The contribution of small deletions, small insertions, SNPs and large deletions to TE variation between Col-0 and Bur-0/C24. (c) Distribution of large deletion sizes within TEs.
(TIF)

Figure S2 TE length distribution by superfamily. Variance of TE length in Bur-0 compared to Col-0 for each TE superfamily.
(TIF)

Figure S3 Depiction of non-standard abbreviations. Cartoon representations of the non-standard abbreviations. Grey regions in the TEs represent variation (large deletions, SNPs and indels), PCG = protein coding gene.
(TIF)

Figure S4 Variant TEs and genes affected by proximal variant TEs. (a) The number of total and variant TEs for Col-0, Bur-0 and C24. The overlap of VarTEs between Col-0 and Bur-0 or C24 is shown in the Venn diagram. (b) The number of TE-, InvTE+ and VarTE+ genes among Col-0, Bur-0 and C24. The overlap of VarTE+ genes between Col-0 and Bur-0 or C24 is shown in the Venn diagram.
(TIF)

Figure S5 Chromosomal distribution of variant TEs in Bur-0. The distribution of total TEs (blue; left y-axis), genes (red; left y-axis), and the percentage of variant TEs (green; right y-axis) for all chromosomes between Col-0 and Bur-0 using a 500 kb sliding window. The black blocks represent the centromeric regions.
(TIF)

Figure S6 Chromosomal distribution of variant TEs in C24. The distribution of total TEs (blue; left y-axis), genes (red; left y-axis), and the percentage of variant TEs (green; right y-axis) for all chromosomes between Col-0 and C24 using a 500 kb sliding window. The black blocks represent the centromeric regions.
(TIF)

Figure S7 Chromosomal distribution of variant TEs. The distribution of total TEs (blue; left y-axis), the percentage of variant distal TEs (red) and proximal TEs (green; right y-axis) for chromosome 1 between Col-0 and Bur-0 using a 500 kb sliding window.
(TIF)

Figure S8 TE variation as a function of neighboring gene distance. Average TE variation by distance to the closest gene for Col-0 vs Bur-0 (blue) or C24 (red). Bin size = 500 bp. MWU p[Col-0/Bur-0] = 0.001, p[Col-0/C24]<6×10^−5^.
(TIF)

Figure S9 Gene polymorphism levels and proximity to TEs for major gene families. Average polymorphism level in the 80 accessions (a) and the three accessions (b; red) and distance to the nearest TE (grey) for major gene families in the three accessions. Spearman's ρ(Col-0) = −0.11, ρ(Bur-0) = −0.11, ρ(C24) = −0.10; p<2×10^−16^.
(TIF)

Figure S10 Gene family and proximal TE frequency. The fraction of genes with proximal TEs for major gene families in each accession (a–c).
(TIF)

Figure S11 TE polymorphism levels with regard to flanking regions and nearby genes. The polymorphism level of TEs and their flanking regions for each TE group [high/low flanking region (FR) polymorphism, high polymorphism/low polymorphism/no genes; Col-0 versus Bur-0/C24] was calculated. Binomial tests between TE groups confirmed significant differences (p = 0) for (a) all polymorphisms and (b) large deletions for: TEs with highly vs lowly polymorphic FRs; TEs with highly vs no or lowly polymorphic flanking genes (with either high or low FR polymorphism. Binomial tests also indicated significance (p = 0) for all polymorphisms (a) and large deletions (b) between TEs vs FRs with the exception of TEs in highly polymorphic regions that contain genes of low polymorphism. (c) Small polymorphisms showed no significant differences between TE groups or between TEs vs FRs.
(TIF)

Figure S12 TE suoerfamilies and neighboring gene expression. Average expression levels for each accession of TE+ genes according to the superfamily of the nearest TE. MWU [retrotransposons vs CACTAs/MITEs] p = 0.02 for Col-0, Bur-0 and C24. MWU [CACTA TE+ genes vs TE− genes] p = 0.7 for Col-0, p = 0.6 for Bur-0 and p = 0.8 for C24). Numbers displayed to the right of the bars indicate statistical groupings (pairwise MWU tests: p<0.05 between groups and p≥0.05 within each group).
(TIF)

Figure S13 siRNA-targeting of non-centromeric TEs. siRNA-targeting of non-centromeric genomic and TE regions in Col-0, Bur-0 and C24. The abundance of siRNA in TEs and genome-wide is defined as the total number of mapped siRNA reads, normalized by total TE and genome length, respectively (see Table S5).
(TIF)

Figure S14 TE superfamilies and siRNA-targeting. The fraction of TEs that are siRNA+ in each TE superfamily for each accession; Col-0 (a), C24 (b), or Bur-0 (c). Binomial test: p = 0 for Col-0, Bur-0 and C24.
(TIF)

Figure S15 Relationship of TE siRNA-targeting to gene proximity and the effect on gene expression in Col-0, Bur-0 and C24. (a) The average distance of siRNA− (red) and siRNA+ (yellow) proximal TEs to the nearest genes. For siRNA+ proximal TEs, distances to the closest gene are compared between msiRNA+ TEs (cyan) and usiRNA+ TEs (navy). (b) Average expression level of genes when neighboring TEs are siRNA− (red) or siRNA+ (yellow). For siRNA+ TEs, average neighboring gene expression levels are given for when the TEs are distal (greater than 2 kb from gene; dark gray) or proximal (within 2 kb; light gray). For genes with proximal siRNA+ TEs, expression levels are further compared for msiRNA+ TEs (cyan) vs usiRNA+ TEs (navy). The number of expressed genes used in each analysis is given. MWU: ** = p<0.01.
(TIF)

Figure S16 siRNA-targeting of TEs and TE proximity to genes by TE superfamily. Average distance to the nearest gene compared between siRNA+ and siRNA− proximal TEs for each TE superfamily for the three accessions (a–c).
(TIF)

Table S1 TE variation by chromosomal position. The number of TEs, average TE variation and fraction of variant TEs between Col-0 and Bur-0/C24 are summarized depending on TE proximity to genes on chromosomes arms and pericentromeric regions. SE = standard error.
(DOCX)

Table S2 TE variation and proximity to genes. The number, average size, average distance to the nearest gene, degree of TE variation, insertion site preference and TE average age summarized by TE superfamily. (*) Rank is presented as descending TE distance to the nearest gene and degree of TE variation (MWU: p-value<0.05). (**) Average age is given for each superfamily where possible. Mean average age for all *A. thaliana* TEs is 11.0 million years [25].
(DOCX)

Table S3 TE and gene numbers for each accession. The number of total and non-centromeric TEs and genes is summarized. The number of genes sorted by TE proximity and TE variation is also given, along with the total number of expressed non-centromeric genes.
(DOCX)

Table S4 siRNA mapping statistics. Twenty-four nt siRNA reads that map to non-centromeric sequences in Col-0, Bur-0 and C24.
(DOCX)

Table S5 siRNA-targeting of TEs. TEs according to siRNA-targeting and siRNA mapping uniqueness. The number of genes is also given according to whether or not the closest TE is targeted by siRNAs.
(DOCX)

Table S6 Gene numbers by polymorphism level and TE presence and variance. Genes categorized by level of genic polymorphism and proximal TE variation.
(DOCX)

Table S7 Candidate genes for TE/siRNA regulation. Genes that are siRNA+ TE+ in Col-0 but siRNA− TE+ or TE− in Bur-0 or C24 and show significant up-regulation (top 5% ranking) in Bur-0 or C24, in addition to at least one RNA silencing mutant.
(DOCX)

Table S8 Invariant and variant TEs targeted by siRNA and their adjacent genes.
(DOCX)
