# Supplementary figures and images for: Transposon Variants and Their Effects on Gene Expression in Arabidopsis
Source: PLoS Genet. 2013 Feb 7;9(2):e1003255. doi: 10.1371/journal.pgen.1003255 (PMC3567156; doi:10.1371/journal.pgen.1003255)

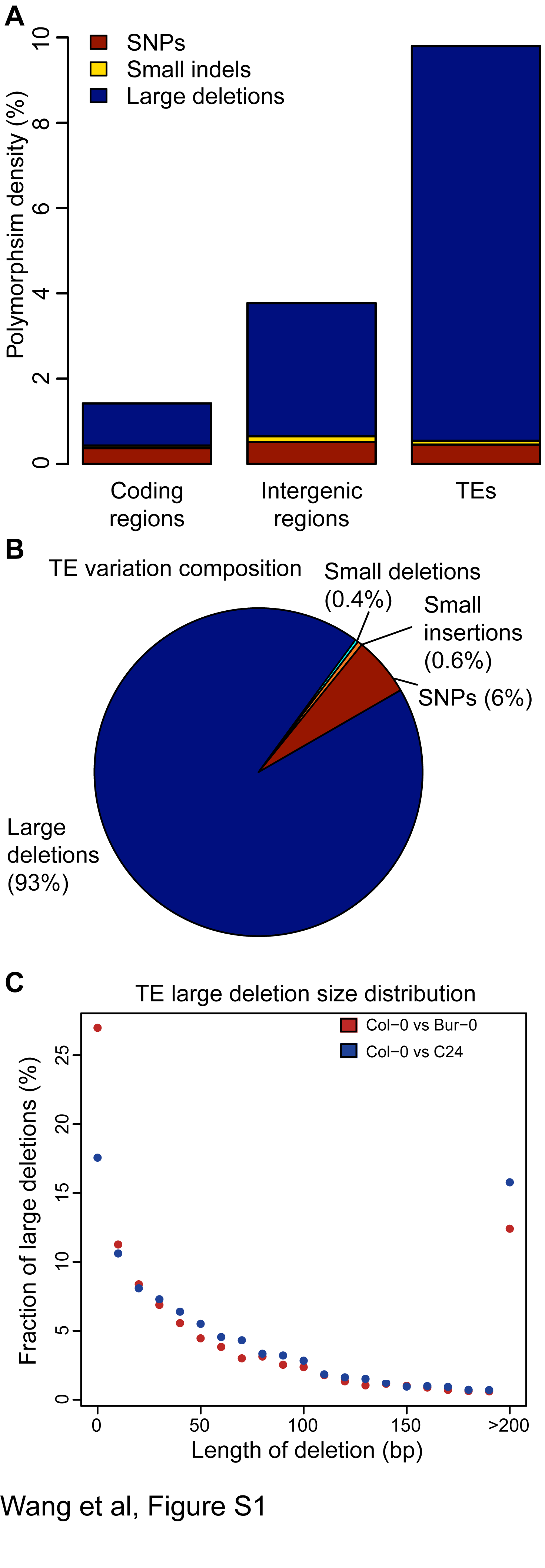

Supplement: Figure S1 — TE variation in Col-0, Bur-0 and C24. (a) Polymorphism densities for coding regions, intergenic regions and TEs according to polymorphism type. Binomial tests: p[Coding Regions/Intergenic Region] = 0 and p[Coding Regions/TE] = 0 for SNPs, indels or large deletions); p[Intergenic Regions/TE] = 0 for large deletions, (b) The contribution of small deletions, small insertions, SNPs and large deletions to TE variation between Col-0 and Bur-0/C24. (c) Distribution of large deletion sizes within TEs. (TIF) [file pgen.1003255.s001.tif]

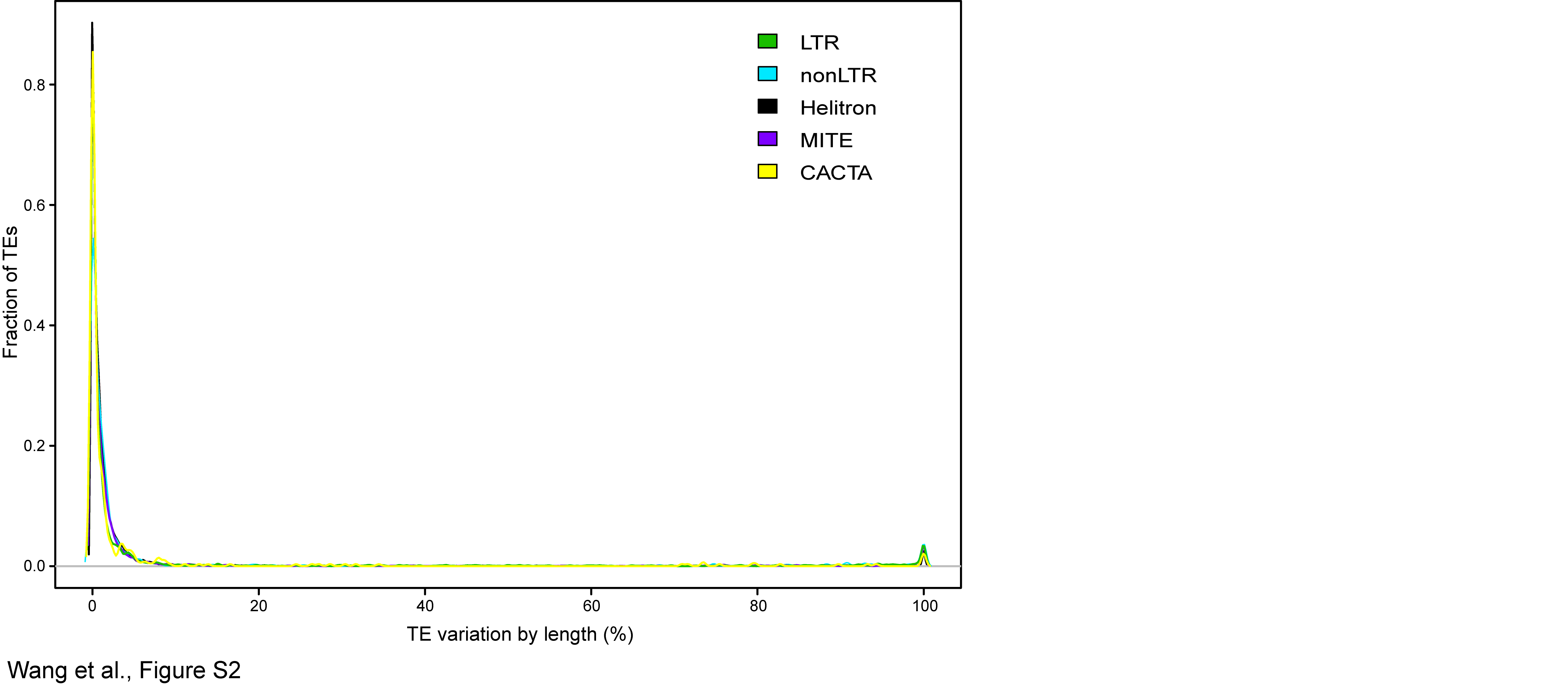

Supplement: Figure S2 — TE length distribution by superfamily. Variance of TE length in Bur-0 compared to Col-0 for each TE superfamily. (TIF) [file pgen.1003255.s002.tif]

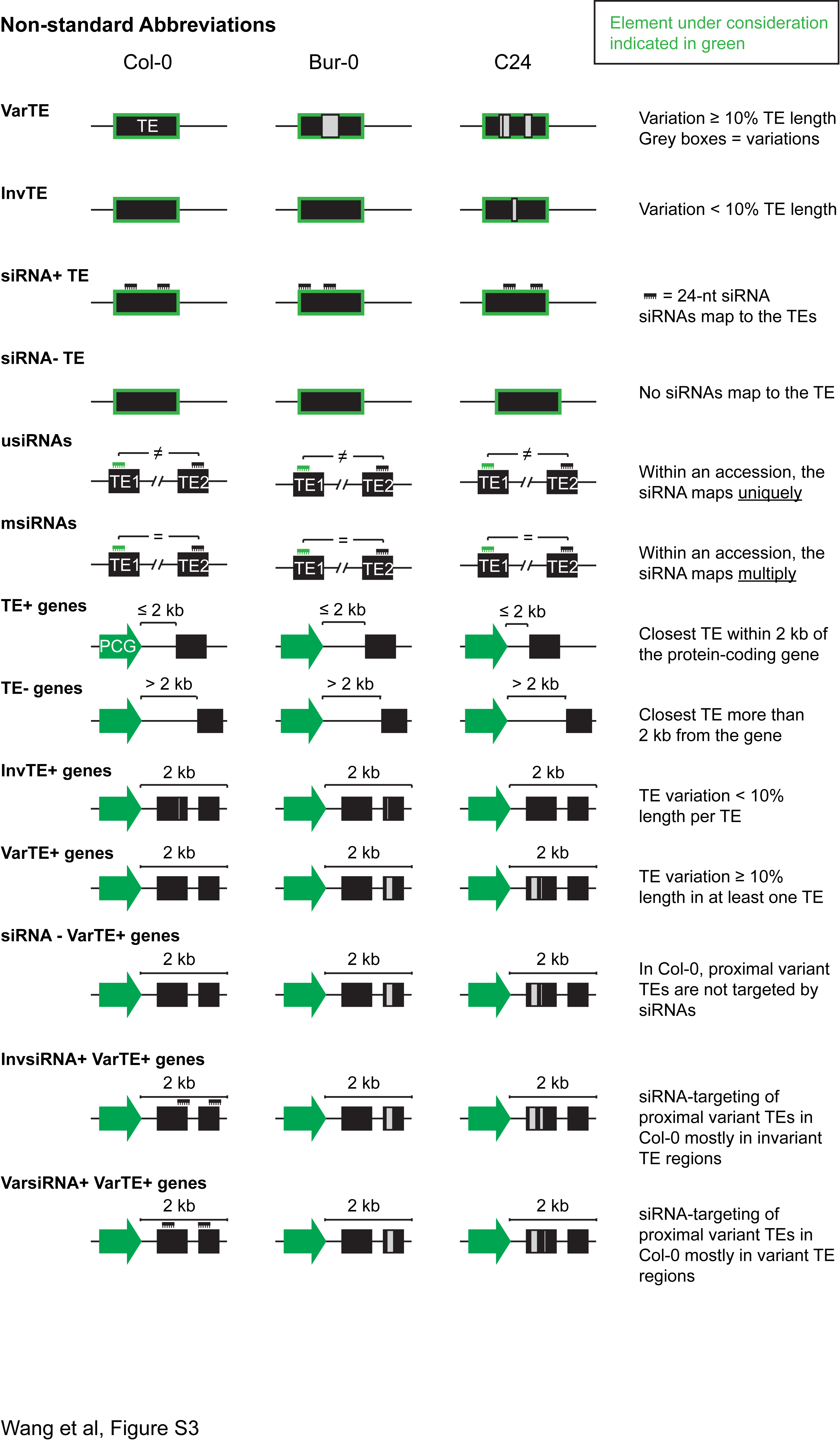

Supplement: Figure S3 — Depiction of non-standard abbreviations. Cartoon representations of the non-standard abbreviations. Grey regions in the TEs represent variation (large deletions, SNPs and indels), PCG = protein coding gene. (TIF) [file pgen.1003255.s003.tif]

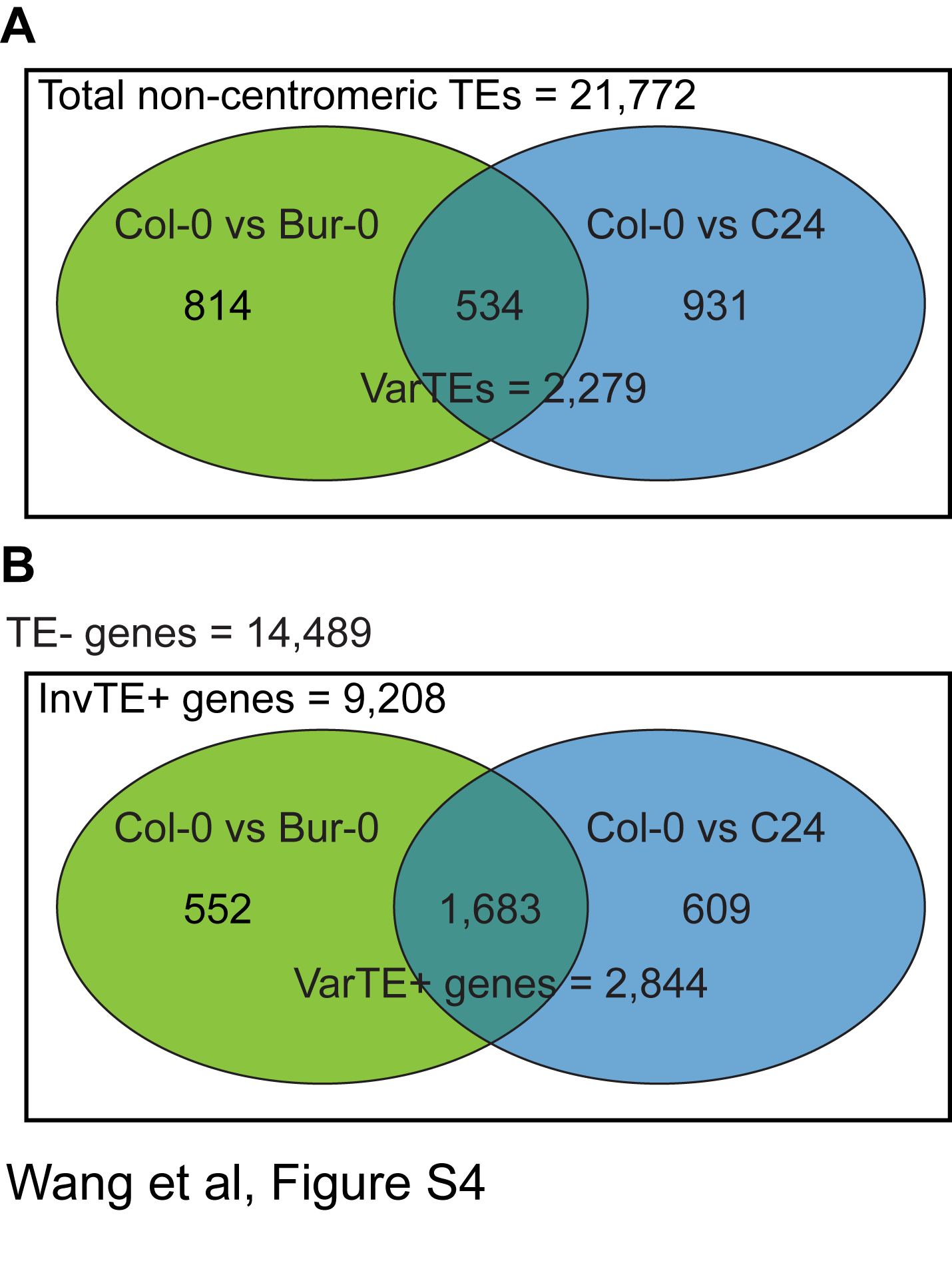

Supplement: Figure S4 — Variant TEs and genes affected by proximal variant TEs. (a) The number of total and variant TEs for Col-0, Bur-0 and C24. The overlap of VarTEs between Col-0 and Bur-0 or C24 is shown in the Venn diagram. (b) The number of TE-, InvTE+ and VarTE+ genes among Col-0, Bur-0 and C24. The overlap of VarTE+ genes between Col-0 and Bur-0 or C24 is shown in the Venn diagram. (TIF) [file pgen.1003255.s004.tif]

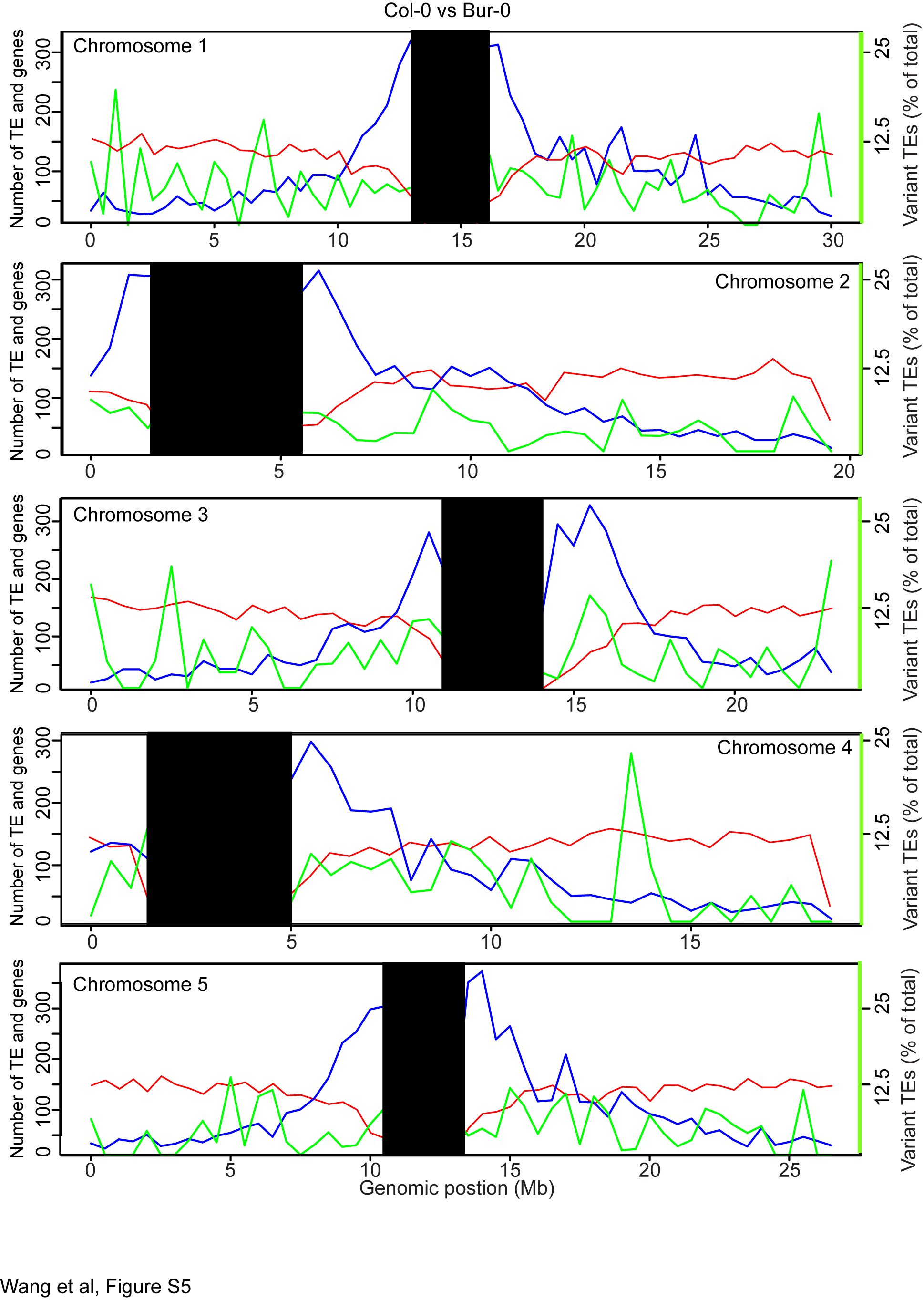

Supplement: Figure S5 — Chromosomal distribution of variant TEs in Bur-0. The distribution of total TEs (blue; left y-axis), genes (red; left y-axis), and the percentage of variant TEs (green; right y-axis) for all chromosomes between Col-0 and Bur-0 using a 500 kb sliding window. The black blocks represent the centromeric regions. (TIF) [file pgen.1003255.s005.tif]

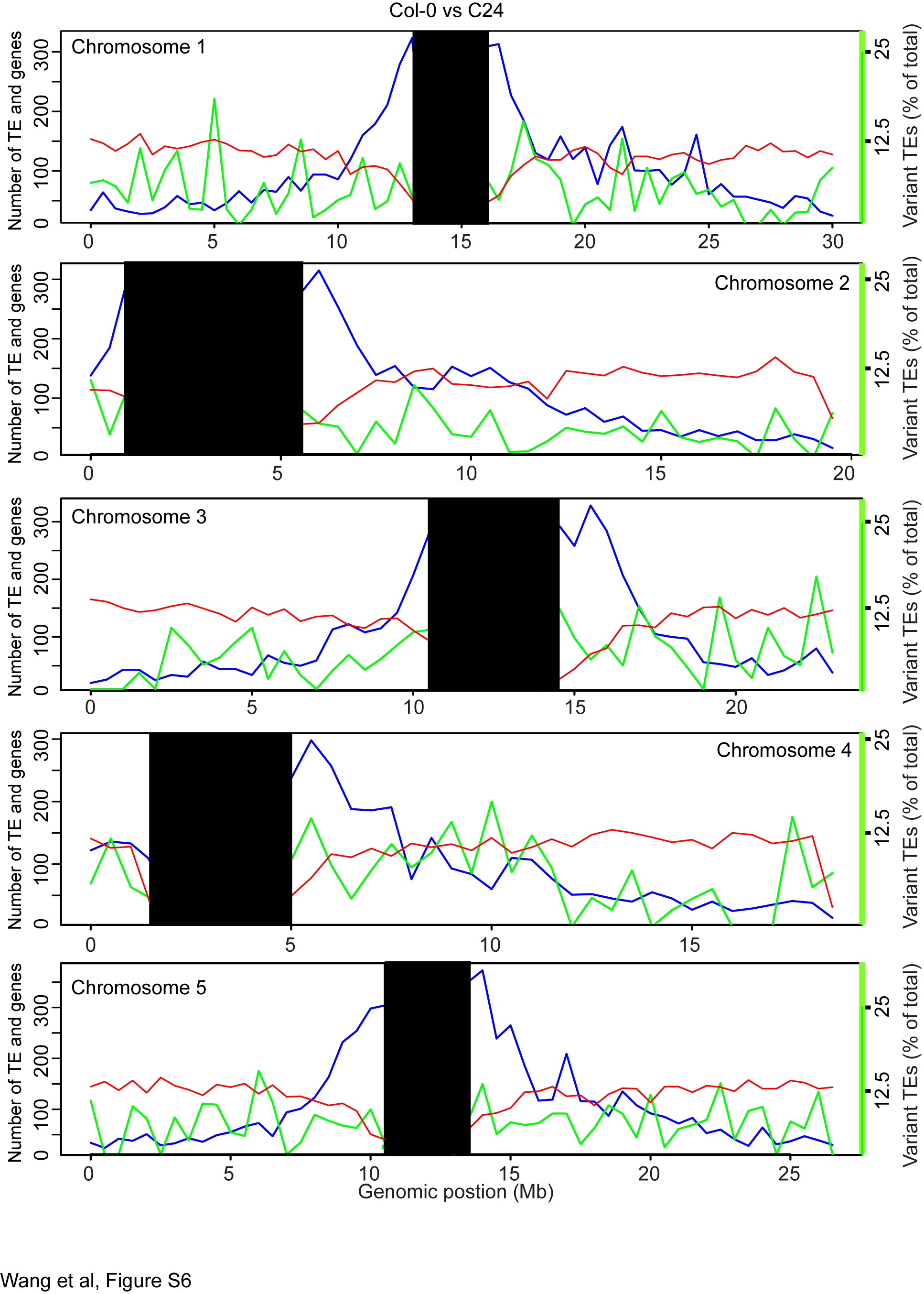

Supplement: Figure S6 — Chromosomal distribution of variant TEs in C24. The distribution of total TEs (blue; left y-axis), genes (red; left y-axis), and the percentage of variant TEs (green; right y-axis) for all chromosomes between Col-0 and C24 using a 500 kb sliding window. The black blocks represent the centromeric regions. (TIF) [file pgen.1003255.s006.tif]

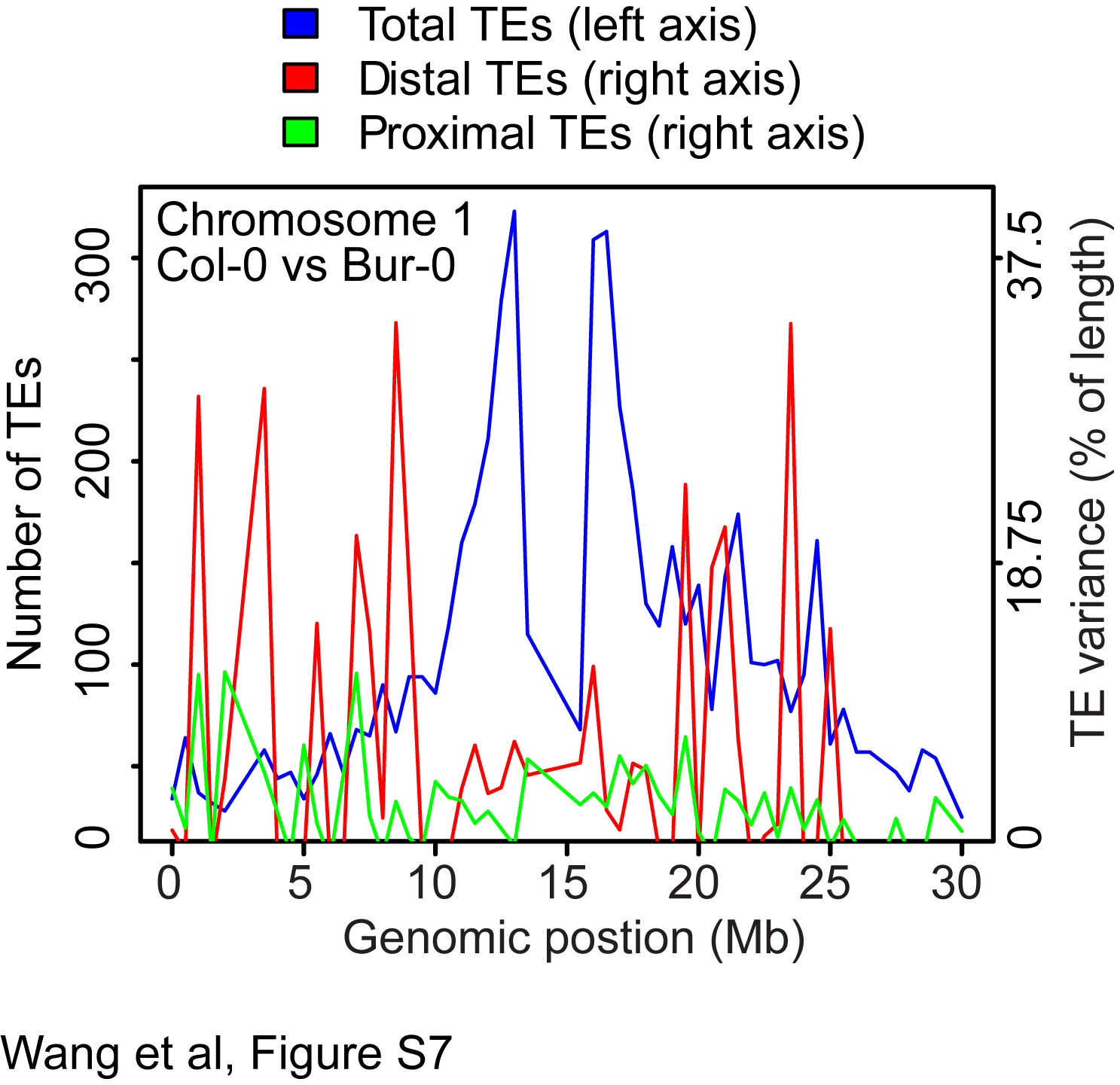

Supplement: Figure S7 — Chromosomal distribution of variant TEs. The distribution of total TEs (blue; left y-axis), the percentage of variant distal TEs (red) and proximal TEs (green; right y-axis) for chromosome 1 between Col-0 and Bur-0 using a 500 kb sliding window. (TIF) [file pgen.1003255.s007.tif]

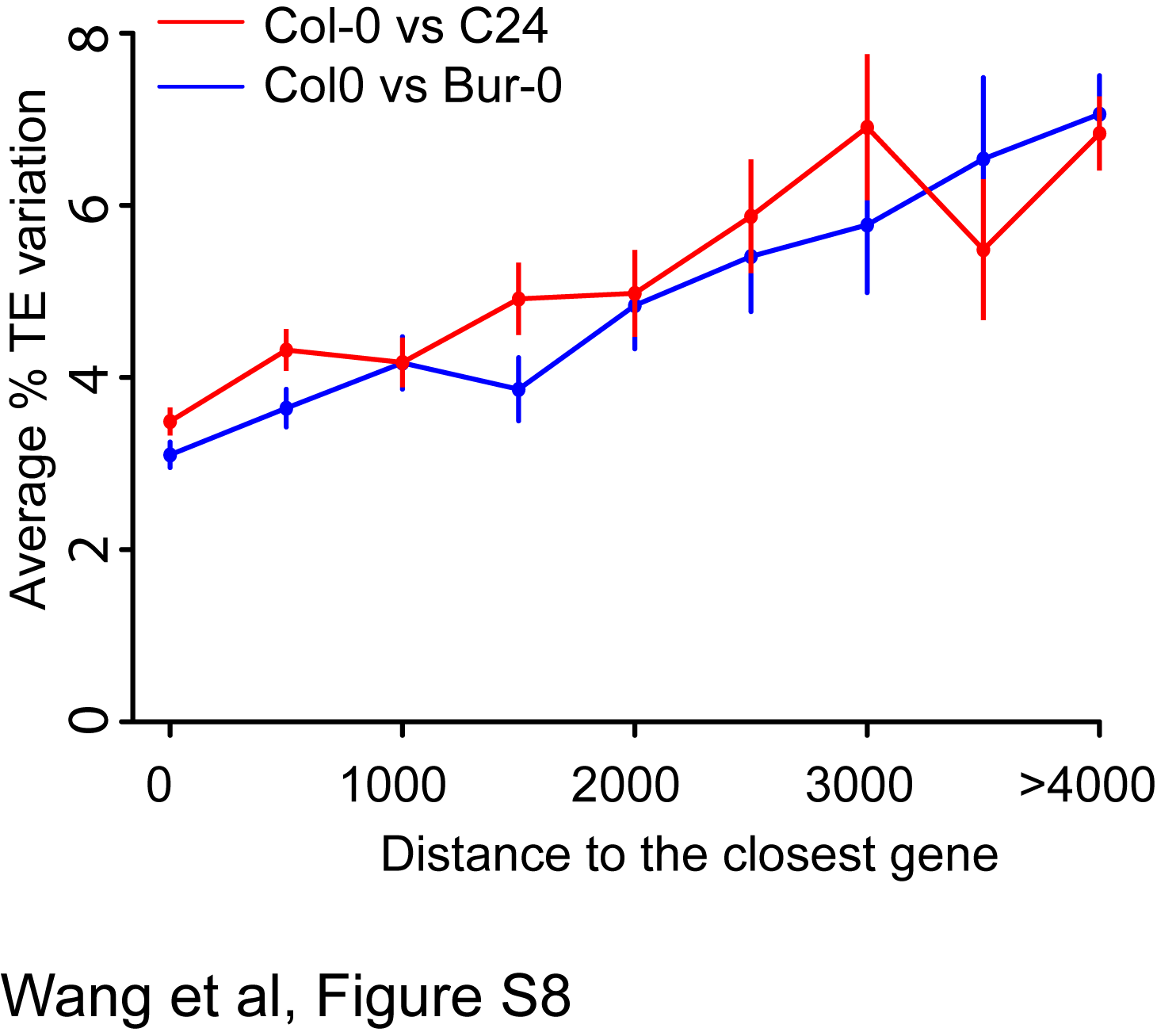

Supplement: Figure S8 — TE variation as a function of neighboring gene distance. Average TE variation by distance to the closest gene for Col-0 vs Bur-0 (blue) or C24 (red). Bin size = 500 bp. MWU p[Col-0/Bur-0] = 0.001, p[Col-0/C24]<6×10^−5^. (TIF) [file pgen.1003255.s008.tif]

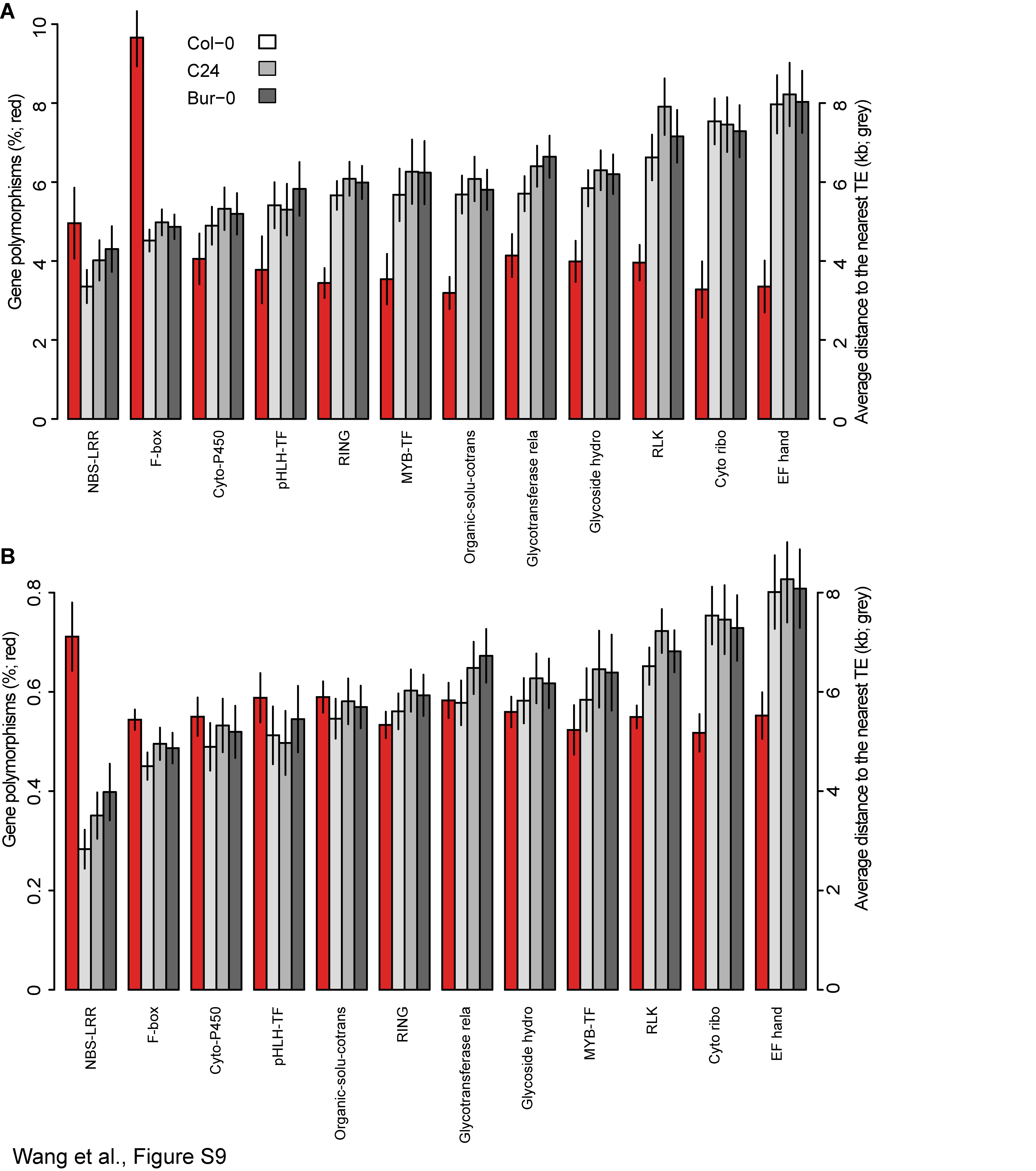

Supplement: Figure S9 — Gene polymorphism levels and proximity to TEs for major gene families. Average polymorphism level in the 80 accessions (a) and the three accessions (b; red) and distance to the nearest TE (grey) for major gene families in the three accessions. Spearman's ρ(Col-0) = −0.11, ρ(Bur-0) = −0.11, ρ(C24) = −0.10; p<2×10^−16^. (TIF) [file pgen.1003255.s009.tif]

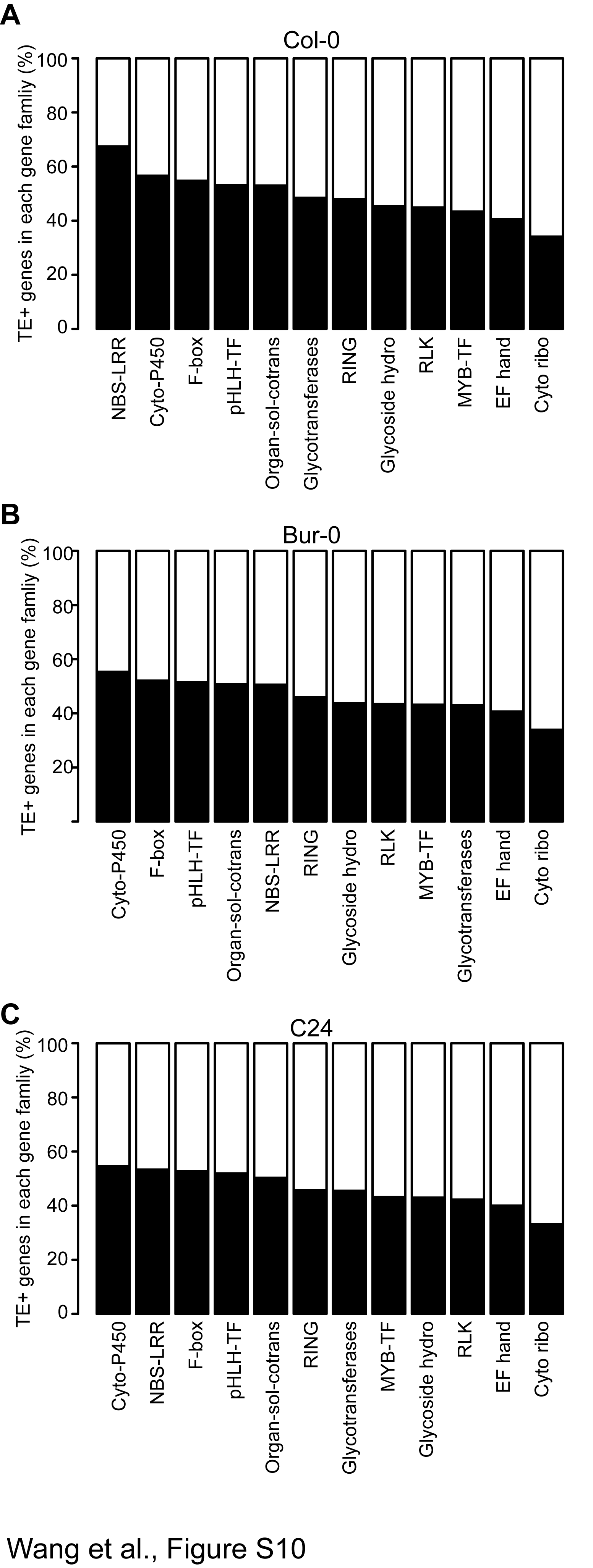

Supplement: Figure S10 — Gene family and proximal TE frequency. The fraction of genes with proximal TEs for major gene families in each accession (a–c). (TIF) [file pgen.1003255.s010.tif]

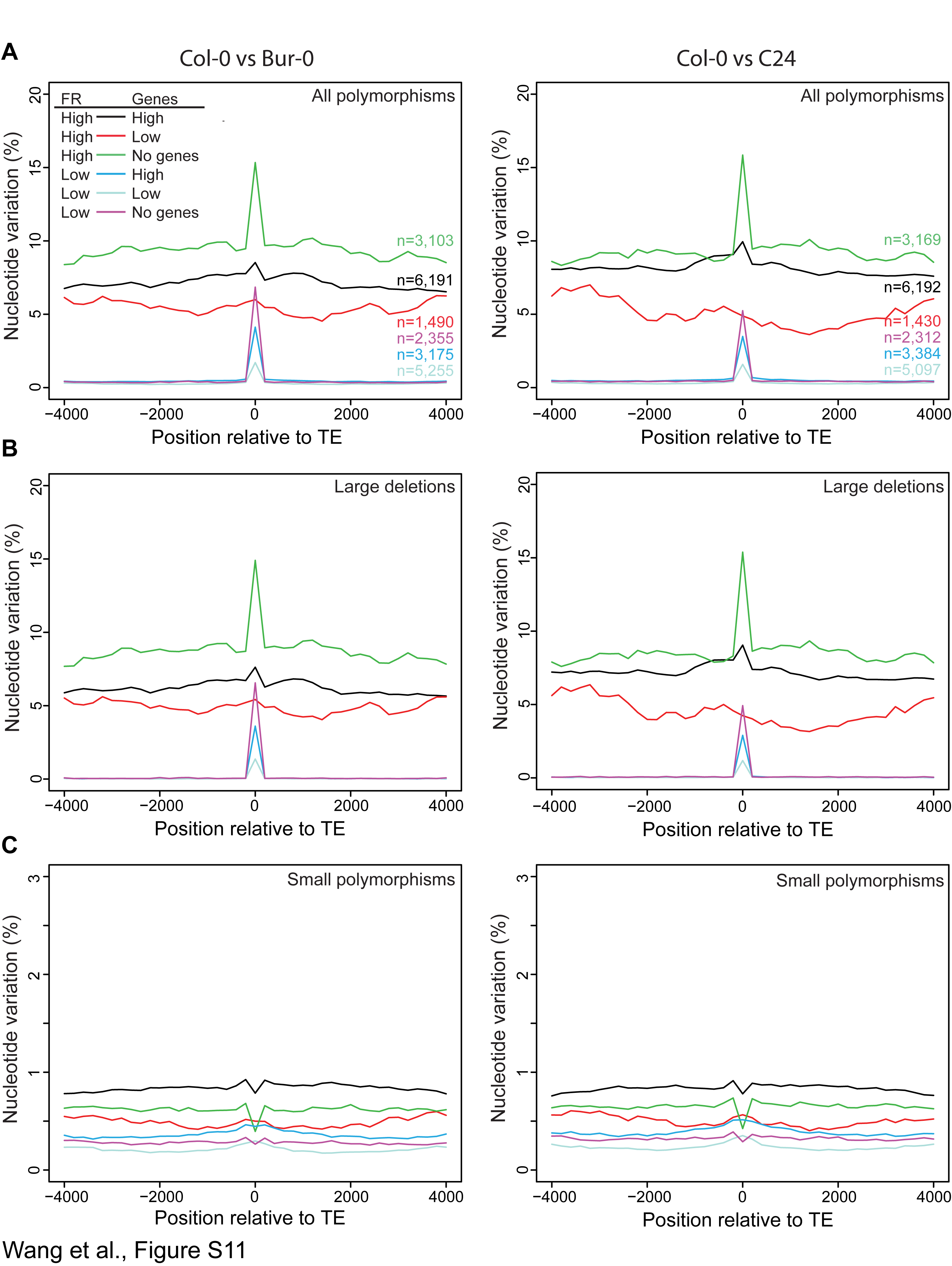

Supplement: Figure S11 — TE polymorphism levels with regard to flanking regions and nearby genes. The polymorphism level of TEs and their flanking regions for each TE group [high/low flanking region (FR) polymorphism, high polymorphism/low polymorphism/no genes; Col-0 versus Bur-0/C24] was calculated. Binomial tests between TE groups confirmed significant differences (p = 0) for (a) all polymorphisms and (b) large deletions for: TEs with highly vs lowly polymorphic FRs; TEs with highly vs no or lowly polymorphic flanking genes (with either high or low FR polymorphism. Binomial tests also indicated significance (p = 0) for all polymorphisms (a) and large deletions (b) between TEs vs FRs with the exception of TEs in highly polymorphic regions that contain genes of low polymorphism. (c) Small polymorphisms showed no significant differences between TE groups or between TEs vs FRs. (TIF) [file pgen.1003255.s011.tif]

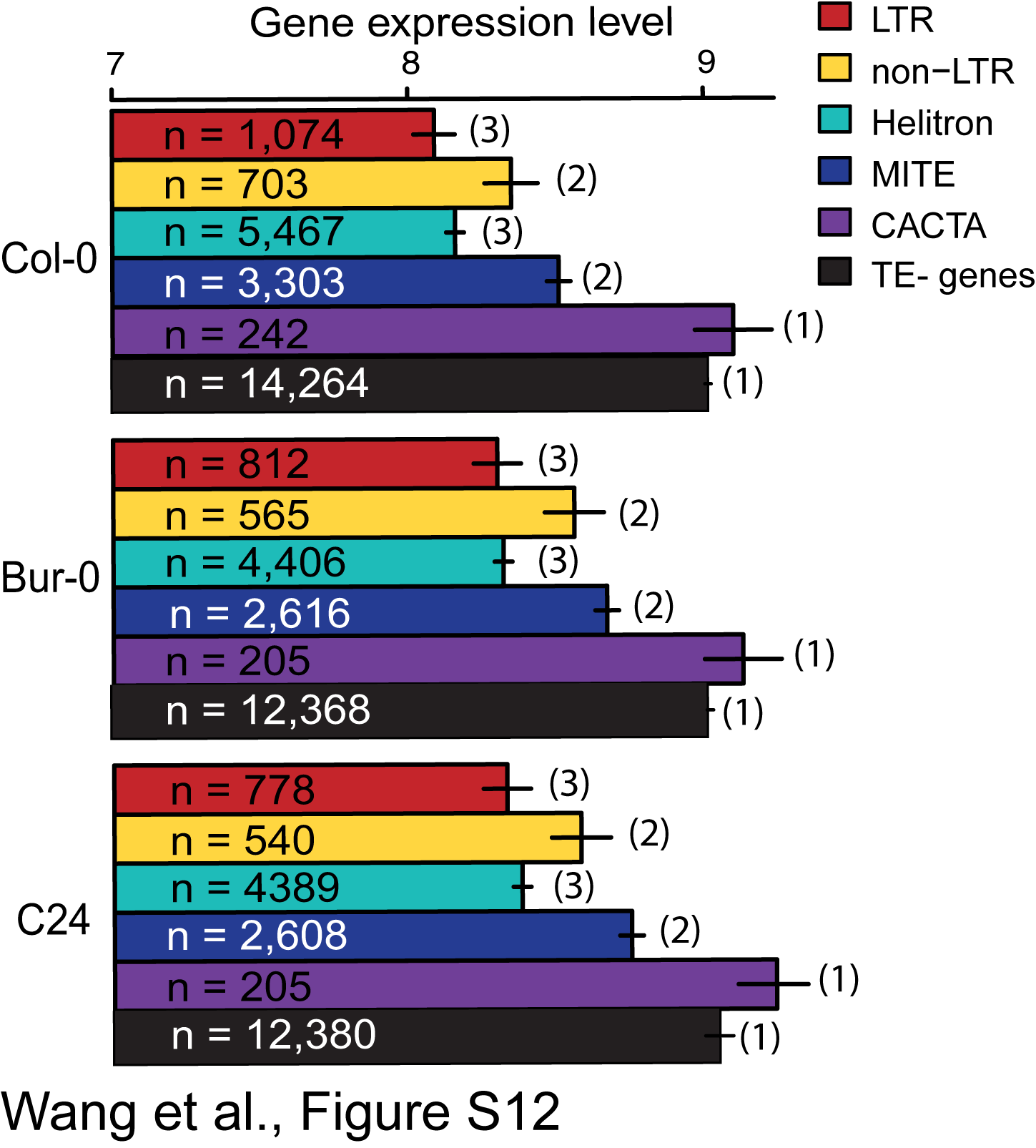

Supplement: Figure S12 — TE suoerfamilies and neighboring gene expression. Average expression levels for each accession of TE+ genes according to the superfamily of the nearest TE. MWU [retrotransposons vs CACTAs/MITEs] p = 0.02 for Col-0, Bur-0 and C24. MWU [CACTA TE+ genes vs TE− genes] p = 0.7 for Col-0, p = 0.6 for Bur-0 and p = 0.8 for C24). Numbers displayed to the right of the bars indicate statistical groupings (pairwise MWU tests: p<0.05 between groups and p≥0.05 within each group). (TIF) [file pgen.1003255.s012.tif]

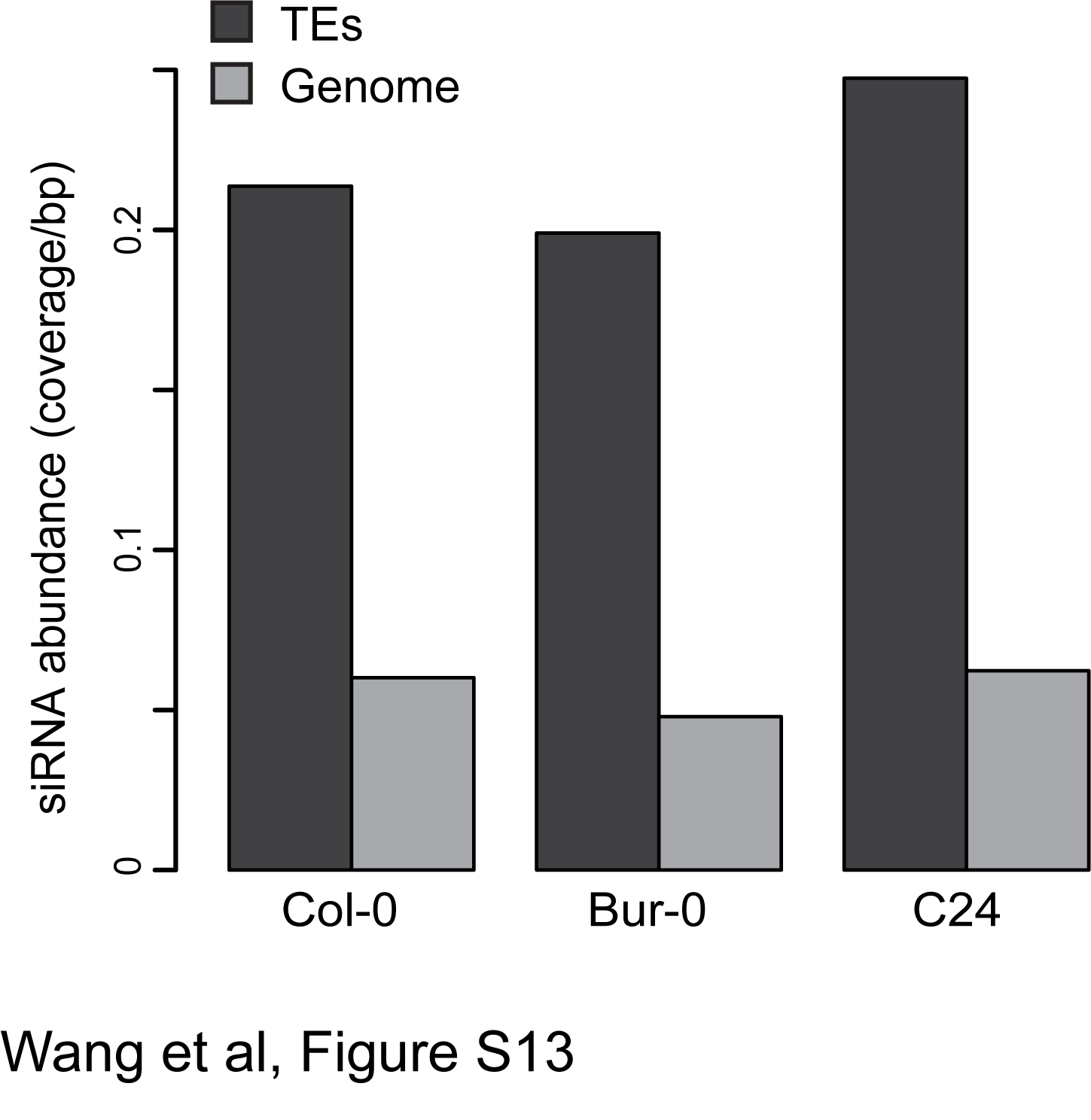

Supplement: Figure S13 — siRNA-targeting of non-centromeric TEs. siRNA-targeting of non-centromeric genomic and TE regions in Col-0, Bur-0 and C24. The abundance of siRNA in TEs and genome-wide is defined as the total number of mapped siRNA reads, normalized by total TE and genome length, respectively (see Table S5). (TIF) [file pgen.1003255.s013.tif]

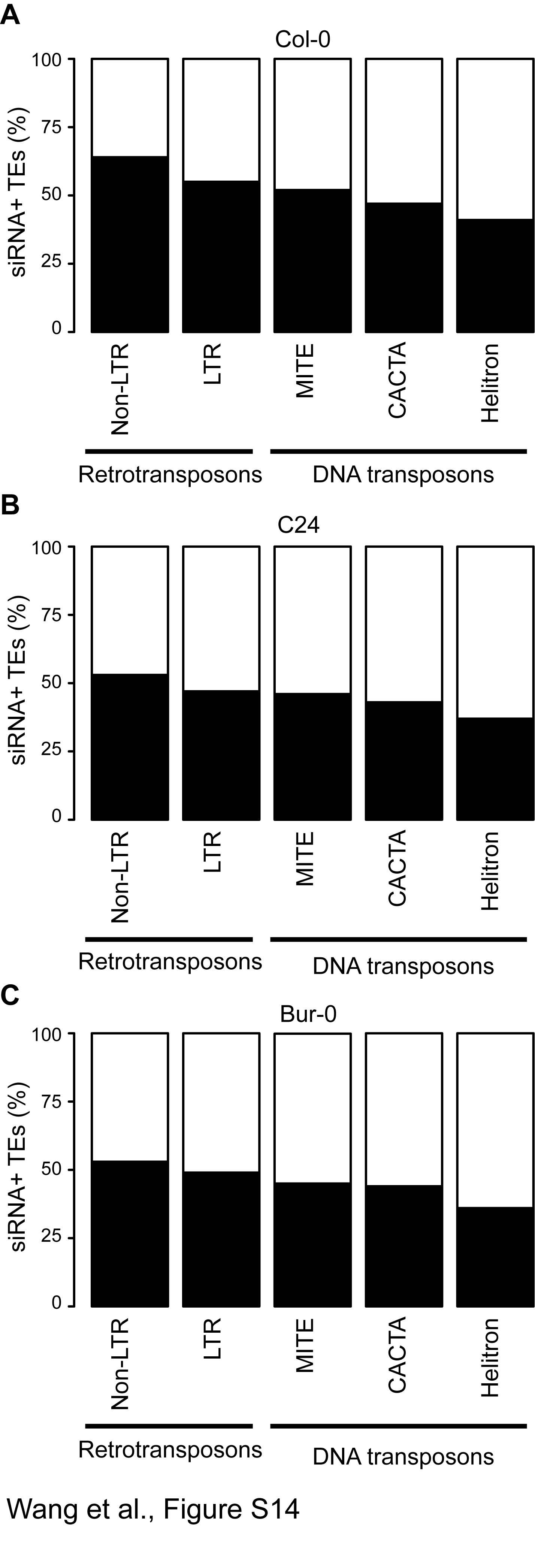

Supplement: Figure S14 — TE superfamilies and siRNA-targeting. The fraction of TEs that are siRNA+ in each TE superfamily for each accession; Col-0 (a), C24 (b), or Bur-0 (c). Binomial test: p = 0 for Col-0, Bur-0 and C24. (TIF) [file pgen.1003255.s014.tif]

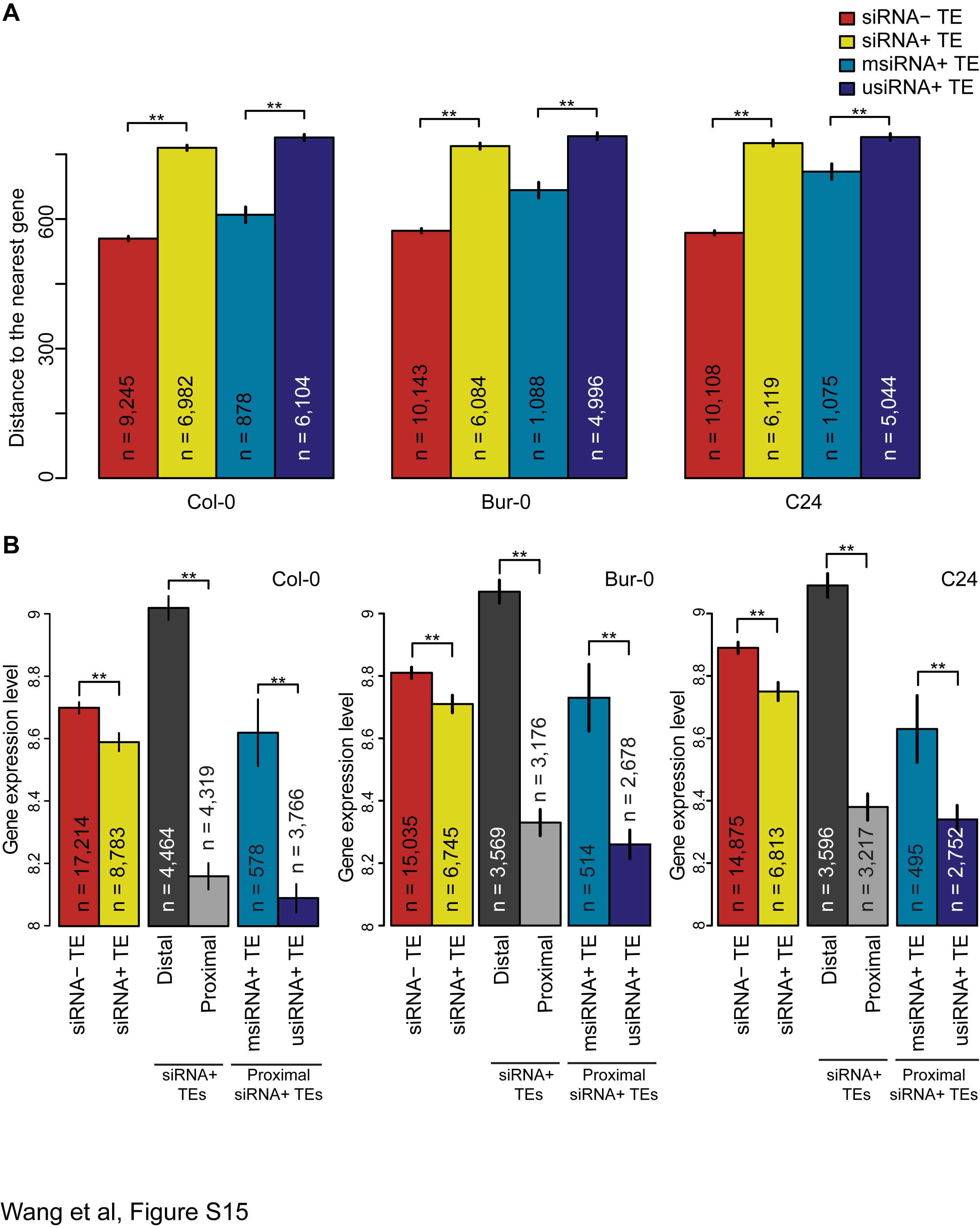

Supplement: Figure S15 — Relationship of TE siRNA-targeting to gene proximity and the effect on gene expression in Col-0, Bur-0 and C24. (a) The average distance of siRNA− (red) and siRNA+ (yellow) proximal TEs to the nearest genes. For siRNA+ proximal TEs, distances to the closest gene are compared between msiRNA+ TEs (cyan) and usiRNA+ TEs (navy). (b) Average expression level of genes when neighboring TEs are siRNA− (red) or siRNA+ (yellow). For siRNA+ TEs, average neighboring gene expression levels are given for when the TEs are distal (greater than 2 kb from gene; dark gray) or proximal (within 2 kb; light gray). For genes with proximal siRNA+ TEs, expression levels are further compared for msiRNA+ TEs (cyan) vs usiRNA+ TEs (navy). The number of expressed genes used in each analysis is given. MWU: ** = p<0.01. (TIF) [file pgen.1003255.s015.tif]

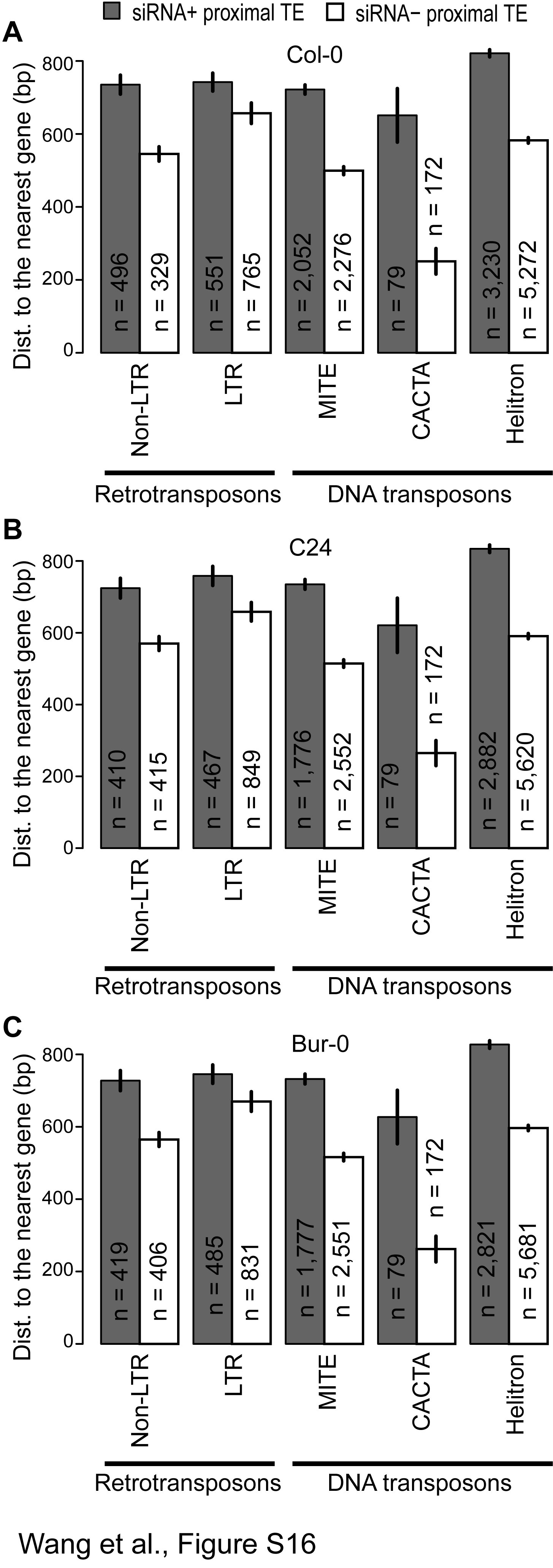

Supplement: Figure S16 — siRNA-targeting of TEs and TE proximity to genes by TE superfamily. Average distance to the nearest gene compared between siRNA+ and siRNA− proximal TEs for each TE superfamily for the three accessions (a–c). (TIF) [file pgen.1003255.s016.tif]
