# Supplementary material for: Transposon Variants and Their Effects on Gene Expression in Arabidopsis
Source: PLoS Genet. 2013 Feb 7;9(2):e1003255. doi: 10.1371/journal.pgen.1003255 (PMC3567156; doi:10.1371/journal.pgen.1003255)
Supplement: Table S1 — TE variation by chromosomal position. The number of TEs, average TE variation and fraction of variant TEs between Col-0 and Bur-0/C24 are summarized depending on TE proximity to genes on chromosomes arms and pericentromeric regions. SE = standard error. (DOCX) [file pgen.1003255.s017.docx]

**Table S1: TE variation by chromosomal position.**

|  | **TE type** | **Number** | **Average variation (%, ± SE)** | | **Fraction of variant TEs (%)** | |
| --- | --- | --- | --- | --- | --- | --- |
|  |  |  | Col-0 vs. Bur-0 | Col-0 vs. C24 | Col-0 vs. Bur-0 | Col-0 vs. C24 |
| **Chromosome arms** | All | 8,097 | 3.5 (±0.16) | 3.7 (±0.16) | 4.6 | 5.1 |
|  | Proximal | 7,082 | 3.2 (±0.16) | 3.4 (±0.16) | 4.1 | 4.6 |
|  | Distal | 1,015 | 6.0 (±0.63) | 6.3 (±0.64) | 7.6 | 8.5 |
| **Peri-centromeric regions** | All | 13,675 | 4.5 (±0.14) | 4.9 (±0.14) | 6.1 | 6.6 |
|  | Proximal | 9,145 | 3.7 (±0.15) | 4.4 (±0.16) | 5.0 | 5.9 |
|  | Distal | 4,530 | 6.2 (±0.29) | 6.1 (±0.29) | 8.3 | 8.1 |

The number of TEs, average TE variation and fraction of variant TEs between Col-0 and Bur-0/C24 are summarized depending on TE proximity to genes on chromosomes arms and pericentromeric regions. SE = standard error.
