# Supplementary material for: Transposon Variants and Their Effects on Gene Expression in Arabidopsis
Source: PLoS Genet. 2013 Feb 7;9(2):e1003255. doi: 10.1371/journal.pgen.1003255 (PMC3567156; doi:10.1371/journal.pgen.1003255)
Supplement: Table S2 — TE variation and proximity to genes. The number, average size, average distance to the nearest gene, degree of TE variation, insertion site preference and TE average age summarized by TE superfamily. (*) Rank is presented as descending TE distance to the nearest gene and degree of TE variation (MWU: p-value<0.05). (**) Average age is given for each superfamily where possible. Mean average age for all A. thaliana TEs is 11.0 million years [25]. (DOCX) [file pgen.1003255.s018.docx]

**Table S2: TE variation and proximity to genes.**

| **Superfamily** | **Number** | **Avg. Size (bp)** | **Avg. distance to nearest gene (bp)** | | **Variable regions (% of total TE Length; median/mean)** | | | **Variant TEs (% for Bur-0/C24)** | **Insertion site preference** | **Average age^**^** |
| --- | --- | --- | --- | --- | --- | --- | --- | --- | --- | --- |
|  |  |  | All TEs (Rank*) | Proximal TE  (Rank*) | Col-0 vs. Bur-0 | Col-0 vs. C24 | Rank* |  |  |  |
| **LTR  retrotransposons** | 2,336 | 811 | 3797 (1) | 624 (3) | 0.00/0.45 | 0.27/0.49 | 3 | 9.2/11.5 | Pericentromere[61] | 3.1 MY[14] |
| **Non-LTR retrotransposons** | 1,183 | 582 | 1849 (2) | 709 (1) | 0.57/0.71 | 0.64/0.73 | 1 | 9.4/10.7 | LINEs and SINEs: gene-rich regions[62,63] |  |
| **CACTAs** | 416 | 671 | 3049 (2) | 376 (4) | 0.00/0.88 | 0.00/0.87 | 4 | 7.0/6.7 | Pericentromere/centromere[64] |  |
| **Helitrons** | 10,975 | 506 | 1571 (2) | 672 (2) | 0.38/0.14 | 0.46/0.14 | 2 | 4.8/4.9 | Pericentromere/centromere[65] | 1-3 MY[65,66] |
| **MITEs** | 5,736 | 436 | 1819 (2) | 606 (3) | 0.25/0.26 | 0.37/0.26 | 3 | 7.3/7.5 | Euchromatics regions[63] |  |

The number, average size, average distance to the nearest gene, degree of TE variation, insertion site preference and TE average age summarized by TE superfamily. (*) Rank is presented as descending TE distance to the nearest gene and degree of TE variation (MWU: p-value<0.05). (**) Average age is given for each superfamily where possible. Mean average age for all *A. thaliana* TEs is 11.0 million years [25].
