# Supplementary material for: Transposon Variants and Their Effects on Gene Expression in Arabidopsis
Source: PLoS Genet. 2013 Feb 7;9(2):e1003255. doi: 10.1371/journal.pgen.1003255 (PMC3567156; doi:10.1371/journal.pgen.1003255)
Supplement: Table S3 — TE and gene numbers for each accession. The number of total and non-centromeric TEs and genes is summarized. The number of genes sorted by TE proximity and TE variation is also given, along with the total number of expressed non-centromeric genes. (DOCX) [file pgen.1003255.s019.docx]

**Table S3: TE and gene numbers for each accession.**

|  | **Col-0** | **Bur-0** | **C24** |
| --- | --- | --- | --- |
| **Total/non-centromeric TEs** | 31,189 / 21,772 | | |
| **Total/non-centromeric genes** | 27,379 / 26,541 | | |
| **TE- genes** | 14,489 | 14,906 | 14,921 |
| **TE+ genes** | 12,052 | 11,635 | 11,620 |
| **Genes with genic, up- and downstream TEs** | 685 | 464 | 439 |
| **Expressed non-centromeric genes** | 25,997 | 21,780 | 21,688 |
| **Expressed TE- genes** | 14,264 | 12,368 | 12,380 |
| **Expressed TE+ genes** | 11,733 | 9,412 | 9,308 |
| **InvTE+ Genes** | 9,208 | | |
| **VarTE+ Genes** | 2,844 | | |

The number of total and non-centromeric TEs and genes is summarized. The number of genes sorted by TE proximity and TE variation is also given, along with the total number of expressed non-centromeric genes.
