# Supplementary material for: Transposon Variants and Their Effects on Gene Expression in Arabidopsis
Source: PLoS Genet. 2013 Feb 7;9(2):e1003255. doi: 10.1371/journal.pgen.1003255 (PMC3567156; doi:10.1371/journal.pgen.1003255)
Supplement: Table S4 — siRNA mapping statistics. Twenty-four nt siRNA reads that map to non-centromeric sequences in Col-0, Bur-0 and C24. (DOCX) [file pgen.1003255.s020.docx]

**Table S4**: **siRNA mapping statistics.**

| **Accession** | **Reads** | **Whole genome** | | | **TE sequences** | | |
| --- | --- | --- | --- | --- | --- | --- | --- |
|  |  | Non-centromeric genome length (bp) | Perfect-match  reads | Unique locations | Non-centromeric total length (bp) | Perfect-match reads | Unique locations |
| **Col-0** | 5,100,648 | 105,717,750 | 3,322,119 | 6,354,808 | 11,377,821 | 989,140 | 2,431,243 |
| **Bur-0** | 7,017,176 | 103,123,175 | 2,439,691 | 4,941,098 | 10,578,260 | 704,306 | 2,105,314 |
| **C24** | 6,141,374 | 102,992,304 | 3,280,799 | 6,414,751 | 10,561,295 | 859,231 | 2,612,833 |

Twenty-four nt siRNA reads that map to non-centromeric sequences in Col-0, Bur-0 and C24.
