# Supplementary material for: Transposon Variants and Their Effects on Gene Expression in Arabidopsis
Source: PLoS Genet. 2013 Feb 7;9(2):e1003255. doi: 10.1371/journal.pgen.1003255 (PMC3567156; doi:10.1371/journal.pgen.1003255)
Supplement: Table S5 — siRNA-targeting of TEs. TEs according to siRNA-targeting and siRNA mapping uniqueness. The number of genes is also given according to whether or not the closest TE is targeted by siRNAs. (DOCX) [file pgen.1003255.s021.docx]

**Table S5**: **siRNA-targeting of TEs.**

|  | **Col-0** | **Bur-0** | **C24** |
| --- | --- | --- | --- |
| **siRNA+ TEs** | 10,424 | 9,093 | 9,139 |
| **Proximal siRNA+ TEs** | 6,982 | 6,084 | 6,119 |
| **siRNA- TEs** | 11,348 | 12,679 | 12,633 |
| **Proximal siRNA- TEs** | 9,245 | 10,143 | 10,108 |
| **usiRNA+ TEs** | 9,141 | 7,512 | 7,562 |
| **Proximal usiRNA+ TEs** | 6,104 | 4,996 | 5,044 |
| **msiRNA+ TEs** | 1,283 | 1,581 | 1,577 |
| **Proximal msiRNA+ TEs** | 878 | 1,088 | 1,075 |
| **siRNA+ TE+ genes** | 9,052 | 8,373 | 8,425 |
| **Expressed siRNA+ TE+ genes** | 8,783 | 6,745 | 6,813 |
| **siRNA- TE+ genes** | 17,489 | 18,168 | 18,116 |
| **Expressed siRNA- TE+ genes** | 17,214 | 15,035 | 14,875 |

TEs according to siRNA-targeting and siRNA mapping uniqueness. The number of genes is also given according to whether or not the closest TE is targeted by siRNAs.
