# Supplementary material for: Transposon Variants and Their Effects on Gene Expression in Arabidopsis
Source: PLoS Genet. 2013 Feb 7;9(2):e1003255. doi: 10.1371/journal.pgen.1003255 (PMC3567156; doi:10.1371/journal.pgen.1003255)
Supplement: Table S6 — Gene numbers by polymorphism level and TE presence and variance. Genes categorized by level of genic polymorphism and proximal TE variation. (DOCX) [file pgen.1003255.s022.docx]

**Table S6**: **Gene numbers by polymorphism level and TE presence and variance**.

|  | **Level of genic polymorphism** | | | |
| --- | --- | --- | --- | --- |
|  | 0-2% | 2-4% | >4% | Total |
| **TE- genes** | 6,519 | 3,753 | 1,314 | 11,586 |
| **InvTE+ genes** | 3,324 | 2,617 | 1,306 | 7,247 |
| **VarTE+ genes** | 697 | 662 | 438 | 1,797 |

Genes categorized by level of genic polymorphism and proximal TE variation.
