# Supplementary material for: Transposon Variants and Their Effects on Gene Expression in Arabidopsis
Source: PLoS Genet. 2013 Feb 7;9(2):e1003255. doi: 10.1371/journal.pgen.1003255 (PMC3567156; doi:10.1371/journal.pgen.1003255)
Supplement: Table S7 — Candidate genes for TE/siRNA regulation. Genes that are siRNA+ TE+ in Col-0 but siRNA− TE+ or TE− in Bur-0 or C24 and show significant up-regulation (top 5% ranking) in Bur-0 or C24, in addition to at least one RNA silencing mutant. (DOCX) [file pgen.1003255.s023.docx]

**Table S7:** **Candidate genes for TE/siRNA regulation**.

| **Gene** | **TE+ in Col-0** | | **siRNA+ in Col-0** | | **Significant change of expression** | | | |
| --- | --- | --- | --- | --- | --- | --- | --- | --- |
|  | TE- in Bur-0 | TE- in C24 | siRNA- in  Bur-0 | siRNA- in C24 | Bur-0/ Col-0 | C24/ Col-0 | *rdr2-1* /WT | *ddc* /WT |
| AT1G31875 |  |  | x | x | x | x | x |  |
| AT1G56510 |  |  | x |  | x |  | x | x |
| AT2G03913 | x | x | x | x | x | x |  | x |
| AT2G16060 |  |  | x |  | x |  | x | x |
| AT2G20465 |  |  | x |  | x |  | x |  |
| AT3G48237 |  |  | x |  | x |  | x |  |
| AT3G62907 |  |  | x |  | x |  | x |  |
| AT4G14400 |  |  | x |  | x |  | x |  |
| AT5G39120 |  |  | x | x | x | x | x |  |
| AT5G46873 |  |  | x |  | x |  | x | x |
| AT5G51190 | x |  | x | x | x | x |  | x |
| AT2G32200 |  |  |  | x |  | x | x | x |
| AT2G32487 |  | x |  | x |  | x | x |  |
| AT3G60570 |  |  |  | x |  | x | x |  |
| AT4G35410 |  | x |  | x |  | x |  | x |

Genes that are siRNA+ TE+ in Col-0 but siRNA- TE+ or TE- in Bur-0 or C24 and show significant up-regulation (top 5% ranking) in Bur-0 or C24, in addition to at least one RNA silencing mutant.
