# Supplementary material for: Transposon Variants and Their Effects on Gene Expression in Arabidopsis
Source: PLoS Genet. 2013 Feb 7;9(2):e1003255. doi: 10.1371/journal.pgen.1003255 (PMC3567156; doi:10.1371/journal.pgen.1003255)
Supplement: Table S8 — Invariant and variant TEs targeted by siRNA and their adjacent genes. (DOCX) [file pgen.1003255.s024.docx]

**Table S8**: **Invariant and variant TEs targeted by siRNA and their adjacent genes**

|  | **Col-0 vs. Bur-0** | **Col-0 vs. C24** |
| --- | --- | --- |
| **siRNA+ InvTEs** | 3,800 | 3,329 |
| **siRNA+ VarTEs*** | 6,731 | 7,202 |
| **InvTE+ genes** | 4,403 | 4,121 |
| **VarTE+ genes*** | 5,924 | 6,206 |
| **siRNA- VarTE+ genes*** | 3,627 | 3,714 |
| **InvsiRNA+ VarTE+ genes*** | 1,084 | 1,084 |
| **VarsiRNA+ VarTE+ genes*** | 271 | 324 |

*VarTEs defined here as TEs with any degree of variation.
